# Supplementary material for: Allosteric regulation and crystallographic fragment screening of SARS-CoV-2 NSP15 endoribonuclease
Source: Nucleic Acids Res. 2023 Apr 28;51(10):5255–70. doi: 10.1093/nar/gkad314 (PMC10250223; doi:10.1093/nar/gkad314)
Supplement: gkad314_Supplemental_Files [file gkad314_supplemental_files.zip › Supplementary datasets.zip!/139/Refine_1/01-BUSTER/Cycle-5/LIST.html]

Refining

**BUSTER 2.X**: A suite of programs for macromolecular structure refinement by maximum-likelihood and Bayesian methods  
Copyright © 1992-2020 Global Phasing Ltd.  
All rights reserved.  
Authors: G. Bricogne, E. Blanc, M. Brandl, C. Flensburg, P. Keller, W. Paciorek, P. Roversi, O. Smart, C. Vonrhein, T. Womack  

Please reference use of the software as:

- BUSTER-TNT 2.X, Global Phasing Ltd, Sheraton House, Cambridge CB3 0AX, UK

Version: 2.10.3 for linux64-ifort made on May 20 2020 at 01:14:34  
Licenced to: Diamond Light Source, Scientific Software  

```
---


### Parsing of the BUSTER cardfile

TITLE : autoBUSTER refinement

   Cell information

   Symmetry information

 Using CCP4 X-ray form factors.

   Missing structure chemical composition

 Using ATOM block for chemical composition.


   Shell information

### Parsing of the TNT cardfile


---


### Reading observations file

 Number of reflexions in input:  82647

      Number of accepted reflexions:  82309

      Number of rejected reflexions:      0

   Preparing complete.mtz containing:      82440 reflexions


   Reflexions in test set:            4081

   Reflexions in working set:        78228


---


### 1. Generating initial model

 2.10 * Shannon sampling rate. NX NY NZ:   360  360  240


  Effective Shannon rates are:    2.50    2.50    2.35


   Allocated BUFFER of size:    119.312 Mb.

   Initial Geometry Information


 No missing atoms declared


   Summary of model volumes

 No missing atoms declared: no prior computed


 Computing bulk solvent model scattering


 NCSMASK PDB radius for solvent mask:     2.15 Angstrom

 running PDBSET (setting cell and spacegroup P1) ... done
 running NCSMASK [1] (creating mask, SMOOTH 0.906025) ... done
 running MAPDUMP (to check if reduction to unit cell is needed) ... done
 running MAPMASK (converting mask to map) ... done

   Generation of babslv by theta-filter

   Electron density in frg mask:  0.420 Å-1

 FOBS  put on rough absolute scale by Wilson plot :
           K        =  0.65356E+02
           B        =     -6.948

 

### Cycle 0001: MaxLik scaling and refinement


   MaxLik Scaling Cycle 0001

   Wilson plot

   Statistics in resolution bins just after scaling
```

|  |  |
| --- | --- |
|  | - Rxpct factors plot- Rxpct factors table- <Log-Lik Gain>- Ln Sigma A- Correlation Coefficients |

```
   <Log-Likelihood> of null-hypothesis

Structure Factor Amplitudes


   Average Figure of Merit

 Switching off refinement of overall temperature factor


 Added B =     0.5926 Å2 to all atoms in fragment


 Added B =     0.5926 Å2 to B_SOLV

```
gelly refinement module
 Authors (2004-2009) O. S. Smart, C. Flensburg, W. Paciorek, C. Vonrhein, 
                     M. Brandl, T. O. Womack and G. Bricogne
         (2010-2011) O. S. Smart, C. Flensburg, W. Paciorek, C. Vonrhein, 
                     T. O. Womack and G. Bricogne
         (2012-2014) O. S. Smart, C. Flensburg, W. Paciorek, C. Vonrhein, 
                     and G. Bricogne
         (2015-2020) G. Bricogne, C. Flensburg, W. Paciorek, C. Vonrhein
 Copyright © 2004-2020 Global Phasing Ltd. 
 All rights reserved.
 gelly module compilation date May 20 2020 01:11:20
 
 gelly uses routines from TNT geometry. References: 
     Tronrud, D. E., Ten Eyck, L. F., Matthews, B. W. (1987)
     "An Efficient General-Purpose Least-Squares Refinement Program 
     for Macromolecular Structures". Acta Crystallogr A, 43:489-501.
     Tronrud, D. E. (1997). "The TNT Refinement Package", 
     in Macromolecular Crystallography, Part B, Eds Charlie Carter 
     and Robert Sweet, Methods in Enzymology, 277:306-319.
 wildcard matching function by Julian Robichaux http://www.nsftools.com

 No -random_seed flag found. Random number stream initialised using:
2147483562
2147483398

 Control flags set to:  -autoncs -hydrogens_input_occ2zero \
                     -hydrogens_output_occ2heavy -screen \
                     100 -screen_sigma \
                     3.0 -keeppdb \
                     1000000 -taomonitor_verbose \
                     2 
 

 ############### Starting setup via TNT routines

 Setting up tnt routines including license check:

                   TNT Stereochemistry Program
 Version: 5.22.3.0
 Licence for Diamond Light Source, Scientific Software is valid
 
 Using TNT geometry routines to load the tnt control file: gelly.tnt
 Using TNT routines to load initial coordinates from file fragment.cor
 Expecting 6119 atoms

 Found     2192 "NOTE BUSTER_RESIDUE_ATOM_DATA" cards
 Have copied information for    6119 atoms from TNT to gelly's store 
 
 The TNT WEIGHT cards used by this run:
 -----WEIGHT       BOND     2.0
 -----WEIGHT       ANGLE    2.0
 -----WEIGHT       IMPROPER 0.0
 -----WEIGHT       TORSION  2.0
 -----WEIGHT       PSEUDO   0.0
 -----WEIGHT       TRIGONAL 2.0
 -----WEIGHT       PLANE    5.0
 -----WEIGHT       CONTACT  5.0
 -----WEIGHT       BCORREL  10.0
 -----WEIGHT       CHIRAL   5.0
 -----WEIGHT       NCS      50.0
 -----WEIGHT       IDEAL    4.0
 -----WEIGHT       RFACTOR  6.13
 
 Dictionary versions picked up from NOTE BUSTER_RESTRAINT_DICT cards as: 
 ------ protgeo_eh99.dat (V1.6.4.17) 20191001  STANDARD AMINO ACID DICTIONARY. BONDS AND ANGLES FROM ENGH AND HUBER EH99. OTHER VALUES BASED ON PREVIOUS TNT OR TAKEN FROM CCP4. INCLUDES HYDROGEN ATOMS.
 ------ exoticaa.dat (V1.3.4.15) 20200327  COLLECTION OF NON-STANDARD AMINO ACIDS, MAINLY EH91 WITHOUT IDEAL DISTANCE INFO
 ------ nuclgeo.dat (V1.13.4.8) 20190909
 ------ bcorrel.dat (V1.15.10.2) 20190616
 ------ contact.dat (V1.15.12.10) 20191121
 ------ idealdist_contact.dat (V1.3.4.12) 20191001  IDEAL-DISTANCE CONTACT TERM DATA AS USED IN PROLSQ. VALUES USED HERE ARE BASED ON DATA HARVESTING OF PDB AS OF 20190109.
 ------ restraints for LIG (Unknown) from cif dictionary Z85934875.cif
 ------ restraints for CIT (CITRIC ACID) from cif dictionary CIT.cif; generated by GRADE_PDB_LIGAND 1.2.12 (October 4 2016) using MOGUL 1.7.2(RC1), CSD as538be, with quantum mechanics RM1 ((DatabaseID PDB-CIT))
 ------ assume.dat (V1.9.4.2) 20180705
 
 Have picked up spacegroup name for pdb output as 'P 63      '
 Setting up Chain/Residue arrays
 
 Have command line specification -autoncs*
     Have found       1 NCS relations
     Pairwise matching residue number and type between chains: 
                    A        B        S    
          A         .       99%       -   
          B                  .        -   
          S                           .   
       Table key--                                                       
          all   : two chains have 100% match and same number of residues 
         multi  : ditto and they contribute to a multi chain ncs cluster 
          ***%  : two chains have >80% matching residue number/type      
           -    : chains appear to be unrelated                          
      chain A     have identified ncs to     1 other chains. So will weight each autoncs by (       6.666/#)=      6.6660
      chain B     have identified ncs to     1 other chains. So will weight each autoncs by (       6.666/#)=      6.6660
      chain S     have identified ncs to     0 other chains. 
     For .Gelly cards produced see below - after pruning
 
 Setting up BUSTER_SET groups
 Have found    89 BUSTER_SET cards
 Now silently applying automatic/dictionary set definitions. For a verbose listing use '-verbose_set' flag.
 Applying user defined set definitions:
 =====NOTE BUSTER_SET ncsautoXcld = Water --> set 'ncsautoXcld' now has   581 atoms 
 =====NOTE BUSTER_SET ncsautoABset = Chain_B \ ncsautoXcld --> set 'ncsautoABset' now has  2770 atoms 
 Have defined    48 sets: 
      set:                 'All' has   6119 atoms in   1281 residues - automatically defined
      set:               'Empty' has      0 atoms in      0 residues - automatically defined
      set:            'ExoticAA' has      0 atoms in      0 residues - defined by dictionary file
      set:           'ExoticNUC' has      0 atoms in      0 residues - defined by dictionary file
      set:              'NotHET' has   5514 atoms in    698 residues - defined by dictionary file
      set:             'Chain_A' has   2768 atoms in    350 residues - automatically defined
      set:             'Chain_B' has   2770 atoms in    350 residues - automatically defined
      set:             'Chain_S' has    581 atoms in    581 residues - automatically defined
      set:               'Water' has    581 atoms in    581 residues - defined by dictionary file
      set:          'StdProtein' has   5514 atoms in    698 residues - defined by dictionary file
      set:             'Protein' has   5514 atoms in    698 residues - defined by dictionary file
      set:                'Back' has   2794 atoms in    698 residues - defined by dictionary file
      set:                'Side' has   2720 atoms in    650 residues - defined by dictionary file
      set:        'RnaCanonical' has      0 atoms in      0 residues - defined by dictionary file
      set:        'DnaCanonical' has      0 atoms in      0 residues - defined by dictionary file
      set:     'RnaDnaCanonical' has      0 atoms in      0 residues - defined by dictionary file
      set:     'DnaRnaCanonical' has      0 atoms in      0 residues - defined by dictionary file
      set:         'RnaExtended' has      0 atoms in      0 residues - defined by dictionary file
      set:         'DnaExtended' has      0 atoms in      0 residues - defined by dictionary file
      set:      'RnaDnaExtended' has      0 atoms in      0 residues - defined by dictionary file
      set:      'DnaRnaExtended' has      0 atoms in      0 residues - defined by dictionary file
      set:                 'Dna' has      0 atoms in      0 residues - defined by dictionary file
      set:                 'Rna' has      0 atoms in      0 residues - defined by dictionary file
      set:              'RnaDna' has      0 atoms in      0 residues - defined by dictionary file
      set:              'DnaRna' has      0 atoms in      0 residues - defined by dictionary file
      set:             'Nucleic' has      0 atoms in      0 residues - defined by dictionary file
      set:             'Polymer' has   5514 atoms in    698 residues - defined by dictionary file
      set: 'BufferWeichenberger' has     13 atoms in      1 residues - defined by dictionary file
      set:    'BufferAdditional' has      0 atoms in      0 residues - defined by dictionary file
      set:              'Buffer' has     13 atoms in      1 residues - defined by dictionary file
      set:         'MetalAlkali' has      0 atoms in      0 residues - defined by dictionary file
      set:  'MetalAlkalineEarth' has      0 atoms in      0 residues - defined by dictionary file
      set:     'MetalTransition' has      0 atoms in      0 residues - defined by dictionary file
      set: 'MetalPostTransition' has      0 atoms in      0 residues - defined by dictionary file
      set:    'MetalLanthanides' has      0 atoms in      0 residues - defined by dictionary file
      set:               'Metal' has      0 atoms in      0 residues - defined by dictionary file
      set:         'SugarAlphaD' has      0 atoms in      0 residues - defined by dictionary file
      set:         'SugarAlphaL' has      0 atoms in      0 residues - defined by dictionary file
      set:               'Sugar' has      0 atoms in      0 residues - defined by dictionary file
      set:               'Other' has     24 atoms in      2 residues - defined by dictionary file
      set:            'Hydrogen' has      0 atoms in      0 residues - defined by dictionary file
      set:         'nonHydrogen' has   6119 atoms in   1281 residues - defined by dictionary file
      set:             'OccZero' has      0 atoms in      0 residues - defined by dictionary file
      set:          'OccNonZero' has   6119 atoms in   1281 residues - defined by dictionary file
      set:             'OccFull' has   6076 atoms in   1278 residues - defined by dictionary file
      set:          'OccNonFull' has     43 atoms in      3 residues - defined by dictionary file
      set:         'ncsautoXcld' has    581 atoms in    581 residues - user specified
      set:        'ncsautoABset' has   2770 atoms in    350 residues - user specified
 No command line arguments '-occ_allhet' or '-occ_restyp' so will not setup automated occcupancy refinement
 
 Interpreting CONSTANT and FREE cards: 
 =====CONSTANT     OCC
 Have found and interpreted      1 CONSTANT cards in the TNT input. 
 Before any COMBINE - Number of atoms with fixed XYZ =     0
 Before any COMBINE - Number of atoms with fixed B   =     0
 Before any COMBINE - Number of atoms with fixed OCC =  6119
 Before any COMBINE - Number of atoms with fixed BIJ =   581
                            cf total number of atoms =  6119
 
 There are no COMBINE cards to be found in the TNT card input 
 
 There are no NOTE BUSTER_COMBINE_RES cards to be found in the TNT card input 

 ############### Working out full connectivity list


 ############### Working out equivalent atoms


 Residue with most atoms given below:
        A|58:N(TRP)         
        A|58:CA(TRP)        
        A|58:CB(TRP)        
        A|58:CG(TRP)        
        A|58:CD1(TRP)       
        A|58:NE1(TRP)       
        A|58:CE2(TRP)       
        A|58:CD2(TRP)       
        A|58:CE3(TRP)       
        A|58:CZ3(TRP)       
        A|58:CH2(TRP)       
        A|58:CZ2(TRP)       
        A|58:C(TRP)         
        A|58:O(TRP)         


 Have no '-equiv_check_plus' argument - so will take atom type into
 account when checking for equivalence of otherwise identical atoms.
 

 ############### Looking at dihedrals


 ############### Looking at chirals

 NOTE BUSTER_CHIRAL_ANGLECUT card found - read value=    -31.00 degs from it
 NOTE BUSTER_CHIRAL_SIGMA card found - read value=      1.70 degs from it
 RANGE allowed for B variables in refinement:   from      3.00000 to    300.00000

 ############### Preparing for PDB output


 ############### Processing extra restraints explicitly defined

 No 'NOTE BUSTER_TARGET' cards or -target?? args found (used to read coordinates files for soft ncs to external)
 No 'NOTE BUSTER_OCCSUM'     cards found - so not using sum of occupancy restraints
 No 'NOTE BUSTER_DISTANCE'   cards found - so not setting up any additional atom-atom distance restraints
 No 'NOTE BUSTER_UTILANGLE'  cards found - so not setting up any additional restraints on any angle
 No 'NOTE BUSTER_UTILTOR'    cards found - so not setting up any additional sinusoidal restraints on torsions
 
 --- Have some similarity definitions 
     Similarity restraints on RMSD use superposition routines from: 
     Coutsias, E.A., Seok, C., Dill, K.A.(2004)
     "Using quaternions to calculate RMSD", J. Comput. Chem., 25:1849-1857 

 Have found    1 'NOTE BUSTER_SIM_DEFINE' cards 
     Maximum number of chains involved in any SIM group (excluding the template)=     1


 ########## Interpreting card: NOTE BUSTER_SIM_DEFINE ncsautoAB ncsautoABset A
     Number of atoms in template set=  2770
     Number of related chains (other than template) in SIM set=    1
     List of chains to be restrained (excluding template)=   A   
     Pairwise RMSD, XYZ in Angstroms, for SIM group: ncsautoAB
                 B         A   
     B         -----     ----- 
     A         0.603     ----- 
     Overall RMSD,XYZ for the SIM group is     0.603 Angstroms
     Pairwise rms deviation of [individual B's minus their set/chain average] in Angs**2
                 B         A         Bavg
     B         -----     -----     50.91
     A         42.34     -----     82.13
     Overall RMSD,B   for the SIM group is    42.340 Angs**2
     The number of common atoms for the above fits:
                 B         A   
     B         -----     ----- 
     A          2757     ----- 
     (compared to the template set that has    2770 atoms in it)
     List of atoms that are absent in one or more chains for this group: 
                                    B    A   
                    C1      401     +    -
                    O1      401     +    -
                    O2      401     +    -
                    C2      401     +    -
                    C3      401     +    -
                    O7      401     +    -
                    C4      401     +    -
                    C5      401     +    -
                    O3      401     +    -
                    O4      401     +    -
                    C6      401     +    -
                    O5      401     +    -
                    O6      401     +    -
     (key  +  atom present for this chain 
           -  atom absent  for this chain)
 transforms for each of the superpositions orthogonal matrix r11 r12 r13 r21 r22 r23 r31 r32 r33 tx ty tz (PDBSET syntax): 
TRANS_BA -0.873968 -0.485122  0.028919 -0.485223  0.874382  0.003880 -0.027168 -0.010641 -0.999574    45.298   -12.543    15.494
 reverse transforms 
TRANS_AB -0.873968 -0.485223 -0.027168 -0.485122  0.874382 -0.010641  0.028919  0.003880 -0.999574    33.924    33.107    14.226
 
 Setting up LSSR interatomic distance restraints
 Interpreting card: NOTE BUSTER_SIM_RESTRAIN_LSSR ncsautoAB 6.666
        setting up interatomic distance pair restraints with:
        Dmin=         0.00
        Dmax=         5.50
        sigma=        0.20
        Vmax=         3.00
        weight=       6.67
        number of distance pairs defined for this set is      34330
 Have not supplied either '-sim_swap_equiv' or '-sim_swap_equiv_plus' argument 
   so will not try swaping equivalent atoms to improve NCS and/or target agreement.
 
 NCS by LSSR outlier analysis
 -autoncs option automatic NCS prune routine -------- 
 Residues pruned out by gradient condition:
          A|147     A|148     A|149     A|150     A|151     A|173     A|181
          A|182     A|185     A|186     A|187     A|188     A|189     A|201
          A|215     A|216     A|217     A|219     A|220     A|223     A|226
          A|229     A|230     A|256     A|259     A|264     A|303     A|306
          A|307     A|328     A|330     A|344     A|345    A|ENDA     B|147
          B|148     B|149     B|150     B|151     B|173     B|180     B|181
          B|182     B|185     B|186     B|187     B|188     B|189     B|201
          B|215     B|216     B|217     B|219     B|220     B|223     B|226
          B|229     B|230     B|256     B|259     B|264     B|303     B|306
          B|307     B|328     B|330     B|344     B|345    B|ENDA
  
 Residues pruned out by function/max condition:
          A|149!    A|187!    A|188!    A|215!    A|264!    A|345!   A|ENDA!
          B|149!    B|187!    B|188!    B|215!    B|264!    B|345!   B|ENDA!
       
 Total number of residues for which LSSR restraints have been pruned out is       69 out of      698 participating
 Number of LSSR restraints pruned out is      4691 this is   13.7% of the total           34330
  
 -autoncs .Gelly cards suitable for adaptation for manual definition/pruning
           (note auto pruning is done in a slightly different way so exact results may differ slightly)
 -autoncs option .Gelly cards also written to file  auto_gelly_cards.txt
NOTE BUSTER_SET ncsautoXcld = Water    # card added - produced by -autoncs
NOTE BUSTER_SET ncsautoXcld = ncsautoXcld + { B|147 B|148 B|149 B|150 B|151 }    # card added - produced by -autoncs
NOTE BUSTER_SET ncsautoXcld = ncsautoXcld + { B|173 B|180 B|181 B|182 B|185 }    # card added - produced by -autoncs
NOTE BUSTER_SET ncsautoXcld = ncsautoXcld + { B|186 B|187 B|188 B|189 B|201 }    # card added - produced by -autoncs
NOTE BUSTER_SET ncsautoXcld = ncsautoXcld + { B|215 B|216 B|217 B|219 B|220 }    # card added - produced by -autoncs
NOTE BUSTER_SET ncsautoXcld = ncsautoXcld + { B|223 B|226 B|229 B|230 B|256 }    # card added - produced by -autoncs
NOTE BUSTER_SET ncsautoXcld = ncsautoXcld + { B|259 B|264 B|303 B|306 B|307 }    # card added - produced by -autoncs
NOTE BUSTER_SET ncsautoXcld = ncsautoXcld + { B|328 B|330 B|344 B|345 B|ENDA }    # card added - produced by -autoncs
NOTE BUSTER_SET ncsautoABset = Chain_B \ ncsautoXcld    # card added - produced by -autoncs
NOTE BUSTER_SIM_DEFINE ncsautoAB ncsautoABset A    # card added - produced by -autoncs
NOTE BUSTER_SIM_RESTRAIN_LSSR ncsautoAB 6.666    # card added - produced by -autoncs

 LSSR function contribution before pruning     34021.7
                             after pruning     10649.7
 Graph of which residues pruned from LSSR: ncs_pruned.mtv
 Graph of LSSR function outliers ncs_function_outliers.mtv
 Graph of LSSR gradient outliers ncs_gradient_outliers.mtv
 LSSR - analysis of distance difference distribution for NCS
         DeltaD less than 0.5 Angs:  proportion of population= 98.71% average DeltaD of subset     0.014 Angs
 Histogram of LSSR deltaD distribution ncs_histogram.mtv
 
 Interpreting card: NOTE BUSTER_TLS_SET tls1 { A|* }
 Interpreting card: NOTE BUSTER_TLS_SET tls2 { B|* }
 Interpreting card: NOTE BUSTER_TLS_SET tls1 { A|* }
 Interpreting card: NOTE BUSTER_TLS_T   tls1     -6.89402E-02  9.73396E-02  1.12143E-02 -5.14314E-02  3.70007E-02 -6.09601E-02
 Interpreting card: NOTE BUSTER_TLS_L   tls1      2.34061E+00  1.26720E+00  1.13030E+00 -7.76197E-01 -1.07598E+00  5.55382E-01
 Interpreting card: NOTE BUSTER_TLS_S   tls1      2.71047E-02 -3.50758E-01  5.52083E-01 -3.65020E-01  2.02419E-01 -2.02943E-01  6.79091E-03 -2.19839E-01
 Interpreting card: NOTE BUSTER_TLS_O   tls1     -2.32263E+01 -8.41675E+01  1.09752E+01
 Interpreting card: NOTE BUSTER_TLS_FIX tls1 ALL
 Interpreting card: NOTE BUSTER_TLS_SET tls2 { B|* }
 Interpreting card: NOTE BUSTER_TLS_T   tls2     -1.17934E-01 -4.34381E-02  3.08544E-02  1.57672E-02  3.71279E-02 -2.24165E-02
 Interpreting card: NOTE BUSTER_TLS_L   tls2      1.51304E+00  9.47989E-01  1.07060E+00  6.77124E-01 -9.34394E-01 -7.04506E-01
 Interpreting card: NOTE BUSTER_TLS_S   tls2     -4.36251E-03  8.11357E-02 -1.53271E-01  1.51330E-01  1.63309E-01  5.90040E-02 -7.47994E-02  3.57312E-02
 Interpreting card: NOTE BUSTER_TLS_O   tls2      2.22079E+01 -7.23190E+01  3.56420E+00
 Interpreting card: NOTE BUSTER_TLS_FIX tls2 ALL


 TLS information. Total number of groups:     2


 Group   Natoms    FixRB   FixTLS   Group Tag    Group Spec
     1     2768      Y        Y     tls1    { A|* }
     2     2770      Y        Y     tls2    { B|* }
 
NOTE BUSTER_TLS_SET tls1 { A|* }
NOTE BUSTER_TLS_T   tls1     -6.89402E-02  9.73396E-02  1.12143E-02 -5.14314E-02  3.70007E-02 -6.09601E-02
NOTE BUSTER_TLS_L   tls1      2.34061E+00  1.26720E+00  1.13030E+00 -7.76197E-01 -1.07598E+00  5.55382E-01
NOTE BUSTER_TLS_S   tls1      2.71047E-02 -3.50758E-01  5.52083E-01 -3.65020E-01  2.02419E-01 -2.02943E-01  6.79091E-03 -2.19839E-01
NOTE BUSTER_TLS_O   tls1     -2.32263E+01 -8.41675E+01  1.09752E+01
NOTE BUSTER_TLS_FIX tls1 ALL
 
NOTE BUSTER_TLS_SET tls2 { B|* }
NOTE BUSTER_TLS_T   tls2     -1.17934E-01 -4.34381E-02  3.08544E-02  1.57672E-02  3.71279E-02 -2.24165E-02
NOTE BUSTER_TLS_L   tls2      1.51304E+00  9.47989E-01  1.07060E+00  6.77124E-01 -9.34394E-01 -7.04506E-01
NOTE BUSTER_TLS_S   tls2     -4.36251E-03  8.11357E-02 -1.53271E-01  1.51330E-01  1.63309E-01  5.90040E-02 -7.47994E-02  3.57312E-02
NOTE BUSTER_TLS_O   tls2      2.22079E+01 -7.23190E+01  3.56420E+00
NOTE BUSTER_TLS_FIX tls2 ALL


 TLS: mmCIF group specification begin


#
loop_
_pdbx_refine_tls_group.id
_pdbx_refine_tls_group.refine_tls_id
_pdbx_refine_tls_group.beg_PDB_ins_code
_pdbx_refine_tls_group.beg_auth_asym_id
_pdbx_refine_tls_group.beg_auth_seq_id
_pdbx_refine_tls_group.end_PDB_ins_code
_pdbx_refine_tls_group.end_auth_asym_id
_pdbx_refine_tls_group.end_auth_seq_id
_pdbx_refine_tls_group.selection
_pdbx_refine_tls_group.selection_details
   1   1 . A   -1 . A  346 . '{ A|* }'
   2   1 . A  501 . A  501 . '{ A|* }'
   3   2 . B   -1 . B  346 . '{ B|* }'
   4   2 . B  401 . B  401 . '{ B|* }'
#


 TLS: mmCIF group specification end


 Interpreting card: NOTE BUSTER_ANISO_SET Empty
 Interpreting card: NOTE BUSTER_ANISO_WEIGHT SCA  16.0
 Interpreting card: NOTE BUSTER_ANISO_WEIGHT SPH   0.6    0.6
 Interpreting card: NOTE BUSTER_ANISO_WEIGHT SIM   7.8    4.8    2.6    0
 Interpreting card: NOTE BUSTER_ANISO_WEIGHT RIG  21.5   21.5    3.7    0


 After TLS and ANISO: Number of atoms with fixed/combined xyz =     0
 After TLS and ANISO: Number of atoms with fixed/combined B   =     0
 After TLS and ANISO: Number of atoms with fixed/combined OCC =  6119
 After TLS and ANISO: Number of atoms with fixed/combined BIJ =  6119
 After TLS and ANISO: Number of atoms in a TLS group          =  5538
                                     cf total number of atoms =  6119
 

 ############### Setup state vector

 No free_vect_ulim_xyz argument found: using default of  100.000
 No free_vect_llim_xyz argument found: using default of -100.000
 Number of parameters added for TLS description                 =       0
 Number of parameters to describe molecular conformation        =   24476

 ############### Setup free vector

 Number of free parameters to be adjusted in refinement   =   24476

 ############### Setting up own geometry


 No command line argument '-tnt_fn' so will use gelly to do geometry function calculations

 ============= Call TNT functions


 ============= Get TNT symmetry information

 
      Getting symmetry operators from TNT. 
          gelly will classify symmetry using pdb-like convention: 
               SYMOP   SYMMETRY
              NNNMMM   OPERATOR
                1555   X,Y,Z
                2555   -Y,X-Y,Z
                3555   -X+Y,-X,Z
                4555   -X,-Y,1/2+Z
                5555   Y,-X+Y,1/2+Z
                6555   X-Y,X,1/2+Z
          where NNN -> operator number and MMM -> translation vector
 

 ============= Own Gelly geometry/restraints setup


 ----------- Identify bond-types


    Setting up GEOMBONDREC_TYPE_HASH* to be able to map a given bond type (e.g.
    "single") to an internal integer (GBOND_TYPE=1) etc.

    Using default  GEOMBONDREC_TYPE_HASH_STR='SING* 1 DOUB* 2 TRIP* 3 AROM* 4 DELO* 4 METAL* 5':
    to override default use a NOTE BUSTER_GEOMBONDREC_TYPE_HASH_STR gelly card.

 ----------- Classify torsion into peptide omega and other


 ----------- Setup of non-bonded terms

 Ideal-distance contact term setup:
    Restraint function constants (normally these values are from NOTE BUSTER_IDEAL_CONTACT_* cards)
    Sigma for general contacts      0.200 Angs
    Sigma for 1-4 contacts          0.100 Angs (contacts across a torsion)
    Sigma for 1-5 contacts          0.150 Angs
    Sigma for 1-N contacts          0.200 Angs
    Sigma for HBOND pairs           0.100 Angs (contacts between HB_types A and D or B's)
    Contact distance increment for each atom in HBOND          -0.150 Angs 
    Contact distance increment for H-bond acceptor              0.100 Angs 
    Contact distance increment for 1-4 with an O atom          -0.050 Angs 
    Contact distance increment for 1-4 with an N atom          -0.180 Angs 
    Contact distance increment for 1-4 with an C atom          -0.370 Angs 
    Contact distance increment for 1-4 with an S atom          -0.150 Angs 
    Contact distance increment for 1-4 with an P atom          -0.150 Angs 
    Contact distance increment for 1-4 with any other atom     -0.150 Angs 
    All atoms matched for CCP4 atom type.
    Number of CCP4 atom types used is         24 (H-bound and not H-bound counted separately)

 Setting up short/ideal-distance contacts
   Using gelly special position handling. (For TNT instead rerun with argument '-special_tnt')

   No command line argument '-special_all' specified. We will auto-generate
   EXCLUDE cards only for atom-atom contacts across symmetry (i.e. on special
   position) when this involves a contact within a residue to itself.

   Have set up non-bonded exclusions - 1-3 contacts
   Atom A|27:CA         makes the most 1-3 contacts:  6

   Have set up non-bonded exclusions - 1-4 contacts
   Atom A|27:N          makes the most 1-4 contacts:  7

   Have set up non-bonded exclusions - 1-5 contacts
   Atom A|28:C.A        makes the most 1-5 contacts: 10

 ############################################################################################################
 ###################################################### setting up contact terms
 ############################################################################################################


 ============= Call Gelly versions of TNT functions

 WARNING: 1-4 contact without 1-(2,3)-4 data for A|501:N06(LIG)       and A|501:N09(LIG)       X-X
 WARNING: 1-4 contact without 1-(2,3)-4 data for A|501:C07(LIG)       and A|501:C10(LIG)       X-X
 WARNING: 1-4 contact without 1-(2,3)-4 data for A|501:C08(LIG)       and A|501:C11(LIG)       X-X
 WARNING: 1-4 contact without 1-(2,3)-4 data for B|401:C1(CIT)        and B|401:C4(CIT)        X-X
 WARNING: 1-4 contact without 1-(2,3)-4 data for B|401:C2(CIT)        and B|401:O6(CIT)        X-X
 WARNING: 1-4 contact without 1-(2,3)-4 data for B|401:O7(CIT)        and B|401:O5(CIT)        X-X
 WARNING: 1-4 contact without 1-(2,3)-4 data for B|401:C4(CIT)        and B|401:O6(CIT)        X-X

       TERM      weight      number      TNT_fn    Gelly_fn        diff
       BOND       2.000        5653    1489.320    1489.320       0.000
       ANGL       2.000        7670    5152.907    5152.907       0.000
       IMPR       0.000           0       0.000       0.000       0.000
       TORS       2.000        1968    2802.843    2802.843      -0.000
       TRIG       2.000           0       0.000       0.000       0.000
       PLAN       5.000         963    2881.405    2881.405       0.000
       CONT       5.000           0     110.859       0.000     110.859
       BCOR      10.000        5630    8355.562    8253.204     102.359


 ============= Call Gelly geometry functions

   Switching torsion functional form to sinusoidal rather than split harmonic functional.
      if you want this not to happen then rerun with '-torsharm'
      Weight for sinusoidal torsion=     2.000
   BCOR function/gradient will be split between H and non-H atoms (as for any atom pair). Use '-bcorrel_all_in_hydrogens' to change this.

 ----------- Fix B-correlation restraints

 
 Checking for bonds that do not have a bcorrel restraint defined
 We will wipe all existing BCORrels if OCC=0.0 atom(s) is/are involved. This can be' switched off via the '-bcorrel_nowipe_zeroocc' flag.
 We will wipe all existing BCORrels if only non-hydrogen atom(s) with fixed B(iso) are involved. This can be switched off via the '-bcorrel_nowipe_fixb_nonhydrogens' flag.
 We will keep all existing BCORrels if hydrogen atom(s) with fixed B(iso) is/are involved. This can be switched off via the '-bcorrel_wipe_fixb_hydrogens' flag.
 No -bcorrel_missing_sigma found. So will set sigma to     2.5 Ang**2 for any bond with no existing BCORrel
 No -bcorrel_missing_sigma_hydrogens found. So will set sigma to     1.0 Ang**2 for any bond with no existing BCORrel involving hydrogens

 No active BCORels found involving OCC=0.0 atom(s)


 No active BCORels found involving fixed-B (only) non-hydrogen atom(s)

    add a default BCOR for bond A|501:C01(LIG) A|501:N02(LIG)
    add a default BCOR for bond A|501:N02(LIG) A|501:C03(LIG)
    add a default BCOR for bond A|501:C03(LIG) A|501:O04(LIG)
    add a default BCOR for bond A|501:C03(LIG) A|501:C05(LIG)
    add a default BCOR for bond A|501:C05(LIG) A|501:N06(LIG)
    add a default BCOR for bond A|501:N06(LIG) A|501:C07(LIG)
    add a default BCOR for bond A|501:N06(LIG) A|501:C11(LIG)
    add a default BCOR for bond A|501:C07(LIG) A|501:C08(LIG)
    add a default BCOR for bond A|501:C08(LIG) A|501:N09(LIG)
    add a default BCOR for bond A|501:N09(LIG) A|501:C10(LIG)
    add a default BCOR for bond A|501:C10(LIG) A|501:C11(LIG)
    add a default BCOR for bond B|401:C1(CIT) B|401:C2(CIT)
    add a default BCOR for bond B|401:C1(CIT) B|401:O1(CIT)
    add a default BCOR for bond B|401:C1(CIT) B|401:O2(CIT)
    add a default BCOR for bond B|401:C2(CIT) B|401:C3(CIT)
    add a default BCOR for bond B|401:C3(CIT) B|401:C4(CIT)
    add a default BCOR for bond B|401:C3(CIT) B|401:C6(CIT)
    add a default BCOR for bond B|401:C3(CIT) B|401:O7(CIT)
    add a default BCOR for bond B|401:C4(CIT) B|401:C5(CIT)
    add a default BCOR for bond B|401:C5(CIT) B|401:O3(CIT)
    add a default BCOR for bond B|401:C5(CIT) B|401:O4(CIT)
    add a default BCOR for bond B|401:C6(CIT) B|401:O5(CIT)
    add a default BCOR for bond B|401:C6(CIT) B|401:O6(CIT)

 Have added a total of        23 BCORrels for bonds which do not already have a BCORrel


 No bonds (for potential BCORrel addition) were skipped due to both atoms having constant/fixed B(iso)


 ----------- Deactivate hydrogen-free planes if hydrogen atoms present

 A total of       0 planes have been deactivated because they are duplicated hydrogen-free and with-hydrogen forms.

 ----------- Handle peptide omega angle(s)

      Torsion angles split into peptide omega and other
         Number of peptide omega angles is       698
         Number of other torsion is             1270
      Peptide omega angles are monitored rather than directly restrained (PLANE used instead).

 ----------- Fix cis-PRO

 Now apply the fix to get correct restraints for cis PRO residues
   Have got      11 bonds records and      26 angles
   from GEOMETRY cards for the special PROC residue
   Weight for ideal-distance contact term=     4.000

 ----------- Prepare SSBOND/LINK for output PDB file


 ----------- Prepare for QM


 ----------- Prepare symmetry in utility distance restraints


 ############### Setting up Rhofit contacts (if needed)

 No ligand-(fixed protein) contact term will be used

 ############### Working out initial function and gradient


   MaxLik Scaling Cycle 0002
 
 Initial functional value = 4.468026E+05 rms gradient=      46.8342
 X-ray contribution to function value = 4.056518E+05
 Geometry contribution (inc ncs)      =      41150.8
 Geometry function summary
     term                           number     weight       rms        GooF      function     fn/numb
     ================================================================================================
     BOND bond lengths (angs)       5653       2.000      0.0073       0.363      1489.32       0.263
     ANGL bond angles (degs)        7670       2.000        0.97       0.580      5152.91       0.672
     TORS split-harmonic tors       1270       0.000       16.70       1.050         0.00       0.000
     SINTOR sinusoidal tors         1270       2.000       16.70                  2138.30       1.684
     PLAN general plane (angs)       963       5.000      0.0155       0.774      2881.40       2.992
     BCORrelations (angs^2)         5653      10.000       1.071       0.382      8253.48       1.460
     CONT (bad contacts)               0       5.000                                 0.00
     IDEAL(ideal-dist contact)      5155       4.000                             10540.34
     CHIRAL (gelly semiharmon)       740       5.000                                45.34
     SIM similarity restraint for NCS and/or TARGET                              10649.67
     OCCSUM restraint on sum of occupanies                                           0.00       as none defined
     DISTAN utility restraints interatomic distance                                  0.00       as none defined
     UTILANGLE utility angle restraints                                              0.00       as none defined
     UTILTOR utility restraints on torsion angles                                    0.00       as none defined
     Peptide omega torsion angles                           3.27 degs - monitor only
 
 WARNING: Have |delta/sigma| deviations > 5.0 sigma. Number of outliers for each term: 
 WARNING:         1 bond lengths. Worst is     5.3 sigs     1.48 Angs A|104:SD=CE (MET)
 WARNING:         1 idealD contacts. Worst     5.0 sigs     2.61 Angs A|104:CE(MET)=S|459:O(HOH) symm: 1555=5555

 Weighted rms      0.0073         0.972  (temporary rms bond and angle for BUSTER to pick up!)
 Weighted intra-residual rms(bond) for StdProtein (excluding H)      0.0071
 Weighted intra-residual rms(bond) for Buffer     (excluding H)      0.0030

 ############### Running initial check


 Initial Geometry Sanity Check

 Check for duplicate bonds: no duplicates found
 
 Check for duplicate or missing bond angles: all are OK
 Check for bond between an atom and itself not involving symmetry:  all are OK
   There are no bad contacts where Delta/sigma is greater than     6.5
   Worst contact dist=   2.605 std=   3.603 sigma=   0.198 abs(Delta/sigma)=    5.040 ats A|104:CE (MET) S|459:O (HOH)
   It is likely that the geometry libraries, sequence info and links match the input molecule
 GEOMETRY CHECK OVERALL:  PASS


 ############### Creating geometry/screen report


 ############### Option "tao" - run Tao optimizer

 TAO: Toolkit for Advanced Optimization. Reference: 
     Munson, T., Sarich, J., Wild, S., Benson, S., McInnes, L.C. (2018)
     "TAO User Manual (Revision 3.9)", Mathematics and Computer Science
     Division, Argonne National Laboratory
     ANL/MCS-TM-322, http://www.mcs.anl.gov/petsc/
 
 Have found -keeppdb flag so will write out a .pdb file every 1000000 its during opt
 Will apply TAO optimization 
 Maximum number of iterations      100
 Convergence test on Grms       4.0000 (-glim)
 Limit on rms displacement to original position (rmsD2init): NONE test inactive (-dlim)

    analysing argument no 0 : -autoncs with j=0
    analysing argument no 1 : -hydrogens_input_occ2zero with j=0
    analysing argument no 2 : -hydrogens_output_occ2heavy with j=0
    analysing argument no 3 : -screen with j=0
    analysing argument no 4 : 100 with j=0
    analysing argument no 5 : -screen_sigma with j=0
    analysing argument no 6 : 3.0 with j=0
    analysing argument no 7 : -keeppdb with j=0
    analysing argument no 8 : 1000000 with j=0
    analysing argument no 9 : -taomonitor_verbose with j=0
    found argument starting with "-tao" : -taomonitor_verbose
    analysing argument no 10 : 2 with j=1
    found value after -tao/-options_/-viewer_ argument : "2"
 PETSC/TAO: setting option "-taomonitor_verbose" = "2"


   MaxLik Scaling Cycle 0003
   Iters     Tot_Funct    Xray_Funct    Geom_Funct          Grms     rmsD2init     maxD2init             R         Rfree           LLG       LLGfree     rms(bond)    rms(angle)       Opttag_H
   Iters             K   B_IMPF_FRAG        K_MISS   B_TEMP_MISS   K_IMPF_MISS   B_IMPF_MISS        K_SOLV   B_TEMP_SOLV   K_IMPF_SOLV   B_IMPF_SOLV        B_TEMP     B_TEMP_11     B_TEMP_12     B_TEMP_22     B_TEMP_13     B_TEMP_23     B_TEMP_33      K_WILSON      B_WILSON      FGCYCLES       Scatag_H
   Iters          BOND         ANGLE      IMPROPER       TORSION    SINTORSION      TRIGONAL        PLANAR  BCORRELATION       CONTACT     IDEALDIST        CHIRAL    SIMILARITY       OCCSSUM  UTILDISTANCE     UTILANGLE   UTILTORSION        RHOFIT        ADP-01        ADP-02        ADP-03        ADP-04        ADP-05        ADP-06        ADP-07        ADP-08        ADP-09        ADP-10      RHOFITPC            QM       Funtag_H
       0  4.467196E+05  4.056520E+05       41067.6       45.4764  0.000000E+00  0.000000E+00      0.203545      0.228608  0.000000E+00  0.000000E+00    0.00728292      0.972401       Opttag_D
       0       73.3126       1.71442       1.00000  0.000000E+00       1.00000  0.000000E+00      0.836296       40.0439       1.00000       44.4427  0.000000E+00       3.91274  0.000000E+00       3.91274  0.000000E+00  0.000000E+00      -7.82548       74.5490       2.25418        2       Scatag_D
       0       1489.32       5152.91  0.000000E+00  0.000000E+00       2138.30  0.000000E+00       2881.40       8170.33  0.000000E+00       10540.3       45.3383       10649.7 -1.000000E+10 -1.000000E+10 -1.000000E+10 -1.000000E+10 -1.000000E+10  0.000000E+00  0.000000E+00  0.000000E+00  0.000000E+00  0.000000E+00  0.000000E+00  0.000000E+00  0.000000E+00  0.000000E+00  0.000000E+00  0.000000E+00  0.000000E+00       Funtag_D
   Geometry Restraint Deviations SCREEN Information

 

 An atom has moved more than     1.50 A from the initial/last
 position - if this was an accepted trial point, probably need to update the
 non-bonded contact list.


   MaxLik Scaling Cycle 0004

   MaxLik Scaling Cycle 0005

   MaxLik Scaling Cycle 0006

   Wilson plot

   Statistics in resolution bins after refinement cycle 0001
```


|  |  |
| --- | --- |
|  | - Rxpct factors plot- Rxpct factors table- <Log-Lik Gain>- Ln Sigma A- Correlation Coefficients |


```
   Iters     Tot_Funct    Xray_Funct    Geom_Funct          Grms     rmsD2init     maxD2init             R         Rfree           LLG       LLGfree     rms(bond)    rms(angle)
   Iters             K   B_IMPF_FRAG        K_MISS   B_TEMP_MISS   K_IMPF_MISS   B_IMPF_MISS        K_SOLV   B_TEMP_SOLV   K_IMPF_SOLV   B_IMPF_SOLV        B_TEMP     B_TEMP_11     B_TEMP_12     B_TEMP_22     B_TEMP_13     B_TEMP_23     B_TEMP_33      K_WILSON      B_WILSON      FGCYCLES
   Iters          BOND         ANGLE      IMPROPER       TORSION    SINTORSION      TRIGONAL        PLANAR  BCORRELATION       CONTACT     IDEALDIST        CHIRAL    SIMILARITY       OCCSSUM  UTILDISTANCE     UTILANGLE   UTILTORSION        RHOFIT        ADP-01        ADP-02        ADP-03        ADP-04        ADP-05        ADP-06        ADP-07        ADP-08        ADP-09        ADP-10      RHOFITPC            QM
       1  4.464369E+05  4.055129E+05       40924.0       27.8033  2.673406E-04    0.00281276      0.203523      0.228621  2.379862E-04 -9.787161E-05    0.00732154      0.972655       Opttag_D
       1       73.3280       1.71331       1.00000  0.000000E+00       1.00000  0.000000E+00      0.836233       40.0763       1.00000       44.4507  0.000000E+00       3.90952  0.000000E+00       3.90952  0.000000E+00  0.000000E+00      -7.81904       74.5490       2.25418        3       Scatag_D
       1       1505.16       5155.59  0.000000E+00  0.000000E+00       2138.06  0.000000E+00       2884.81       8003.80  0.000000E+00       10542.4       45.2862       10648.9 -1.000000E+10 -1.000000E+10 -1.000000E+10 -1.000000E+10 -1.000000E+10  0.000000E+00  0.000000E+00  0.000000E+00  0.000000E+00  0.000000E+00  0.000000E+00  0.000000E+00  0.000000E+00  0.000000E+00  0.000000E+00  0.000000E+00  0.000000E+00       Funtag_D
   Geometry Restraint Deviations SCREEN Information

 

   MaxLik Scaling Cycle 0007

   Wilson plot

   Statistics in resolution bins after refinement cycle 0002
```


|  |  |
| --- | --- |
|  | - Rxpct factors plot- Rxpct factors table- <Log-Lik Gain>- Ln Sigma A- Correlation Coefficients |


```
   Iters     Tot_Funct    Xray_Funct    Geom_Funct          Grms     rmsD2init     maxD2init             R         Rfree           LLG       LLGfree     rms(bond)    rms(angle)
   Iters             K   B_IMPF_FRAG        K_MISS   B_TEMP_MISS   K_IMPF_MISS   B_IMPF_MISS        K_SOLV   B_TEMP_SOLV   K_IMPF_SOLV   B_IMPF_SOLV        B_TEMP     B_TEMP_11     B_TEMP_12     B_TEMP_22     B_TEMP_13     B_TEMP_23     B_TEMP_33      K_WILSON      B_WILSON      FGCYCLES
   Iters          BOND         ANGLE      IMPROPER       TORSION    SINTORSION      TRIGONAL        PLANAR  BCORRELATION       CONTACT     IDEALDIST        CHIRAL    SIMILARITY       OCCSSUM  UTILDISTANCE     UTILANGLE   UTILTORSION        RHOFIT        ADP-01        ADP-02        ADP-03        ADP-04        ADP-05        ADP-06        ADP-07        ADP-08        ADP-09        ADP-10      RHOFITPC            QM
       2  4.463009E+05  4.052326E+05       41068.3       31.5415  8.076691E-04    0.00536282      0.203353      0.228500  8.225156E-04  5.192699E-05    0.00743742      0.974217       Opttag_D
       2       73.3674       1.71292       1.00000  0.000000E+00       1.00000  0.000000E+00      0.836174       40.1640       1.00000       44.4452  0.000000E+00       3.90234  0.000000E+00       3.90234  0.000000E+00  0.000000E+00      -7.80468       74.5490       2.25418        1       Scatag_D
       2       1553.18       5172.16  0.000000E+00  0.000000E+00       2137.60  0.000000E+00       2895.51       8063.26  0.000000E+00       10548.8       45.4850       10652.3 -1.000000E+10 -1.000000E+10 -1.000000E+10 -1.000000E+10 -1.000000E+10  0.000000E+00  0.000000E+00  0.000000E+00  0.000000E+00  0.000000E+00  0.000000E+00  0.000000E+00  0.000000E+00  0.000000E+00  0.000000E+00  0.000000E+00  0.000000E+00       Funtag_D
   Geometry Restraint Deviations SCREEN Information

 
       3  4.462125E+05  4.050688E+05       41143.7       27.1742    0.00120444    0.00856100      0.203240      0.228418    0.00116407  1.583534E-04    0.00750088      0.974860       Opttag_D
       3       73.3955       1.71364       1.00000  0.000000E+00       1.00000  0.000000E+00      0.836170       40.2266       1.00000       44.4298  0.000000E+00       3.89814  0.000000E+00       3.89814  0.000000E+00  0.000000E+00      -7.79629       74.5490       2.25418        1       Scatag_D
       3       1579.80       5179.00  0.000000E+00  0.000000E+00       2137.14  0.000000E+00       2904.88       8085.85  0.000000E+00       10554.6       45.7124       10656.7 -1.000000E+10 -1.000000E+10 -1.000000E+10 -1.000000E+10 -1.000000E+10  0.000000E+00  0.000000E+00  0.000000E+00  0.000000E+00  0.000000E+00  0.000000E+00  0.000000E+00  0.000000E+00  0.000000E+00  0.000000E+00  0.000000E+00  0.000000E+00       Funtag_D
       4  4.461395E+05  4.048897E+05       41249.8       24.1243    0.00169076     0.0130413      0.203106      0.228322    0.00153757  2.720783E-04    0.00756841      0.975568       Opttag_D
       4       73.4302       1.71512       1.00000  0.000000E+00       1.00000  0.000000E+00      0.836178       40.3042       1.00000       44.4072  0.000000E+00       3.89329  0.000000E+00       3.89329  0.000000E+00  0.000000E+00      -7.78659       74.5490       2.25418        1       Scatag_D
       4       1608.37       5186.53  0.000000E+00  0.000000E+00       2136.62  0.000000E+00       2917.00       8128.72  0.000000E+00       10561.9       45.9845       10664.7 -1.000000E+10 -1.000000E+10 -1.000000E+10 -1.000000E+10 -1.000000E+10  0.000000E+00  0.000000E+00  0.000000E+00  0.000000E+00  0.000000E+00  0.000000E+00  0.000000E+00  0.000000E+00  0.000000E+00  0.000000E+00  0.000000E+00  0.000000E+00       Funtag_D
       5  4.460669E+05  4.046537E+05       41413.1       23.2994    0.00240643     0.0202148      0.202922      0.228175    0.00202972  4.593266E-04    0.00764443      0.977447       Opttag_D
       5       73.4818       1.71797       1.00000  0.000000E+00       1.00000  0.000000E+00      0.836203       40.4174       1.00000       44.3719  0.000000E+00       3.88656  0.000000E+00       3.88656  0.000000E+00  0.000000E+00      -7.77313       74.5490       2.25418        1       Scatag_D
       5       1640.84       5206.52  0.000000E+00  0.000000E+00       2135.87  0.000000E+00       2935.57       8195.34  0.000000E+00       10573.7       46.3498       10679.0 -1.000000E+10 -1.000000E+10 -1.000000E+10 -1.000000E+10 -1.000000E+10  0.000000E+00  0.000000E+00  0.000000E+00  0.000000E+00  0.000000E+00  0.000000E+00  0.000000E+00  0.000000E+00  0.000000E+00  0.000000E+00  0.000000E+00  0.000000E+00       Funtag_D
       6  4.460023E+05  4.044237E+05       41578.6       23.5864    0.00323793     0.0295655      0.202726      0.227996    0.00250929  7.060994E-04    0.00770368      0.979620       Opttag_D
       6       73.5421       1.72215       1.00000  0.000000E+00       1.00000  0.000000E+00      0.836256       40.5430       1.00000       44.3289  0.000000E+00       3.87936  0.000000E+00       3.87936  0.000000E+00  0.000000E+00      -7.75872       74.5490       2.25418        1       Scatag_D
       6       1666.38       5229.69  0.000000E+00  0.000000E+00       2135.02  0.000000E+00       2957.74       8259.13  0.000000E+00       10587.9       46.7069       10696.0 -1.000000E+10 -1.000000E+10 -1.000000E+10 -1.000000E+10 -1.000000E+10  0.000000E+00  0.000000E+00  0.000000E+00  0.000000E+00  0.000000E+00  0.000000E+00  0.000000E+00  0.000000E+00  0.000000E+00  0.000000E+00  0.000000E+00  0.000000E+00       Funtag_D
       7  4.459452E+05  4.043194E+05       41625.9       19.2502    0.00375953     0.0365229      0.202623      0.227880    0.00272690  8.780328E-04    0.00770497      0.980264       Opttag_D
       7       73.5797       1.72546       1.00000  0.000000E+00       1.00000  0.000000E+00      0.836306       40.6144       1.00000       44.3005  0.000000E+00       3.87560  0.000000E+00       3.87560  0.000000E+00  0.000000E+00      -7.75120       74.5490       2.25418        1       Scatag_D
       7       1666.94       5236.58  0.000000E+00  0.000000E+00       2134.50  0.000000E+00       2971.04       8269.48  0.000000E+00       10597.3       46.8630       10703.2 -1.000000E+10 -1.000000E+10 -1.000000E+10 -1.000000E+10 -1.000000E+10  0.000000E+00  0.000000E+00  0.000000E+00  0.000000E+00  0.000000E+00  0.000000E+00  0.000000E+00  0.000000E+00  0.000000E+00  0.000000E+00  0.000000E+00  0.000000E+00       Funtag_D
       8  4.458936E+05  4.042423E+05       41651.2       18.6276    0.00430150     0.0446498      0.202534      0.227761    0.00288759    0.00106205    0.00768995      0.981308       Opttag_D
       8       73.6179       1.72919       1.00000  0.000000E+00       1.00000  0.000000E+00      0.836366       40.6791       1.00000       44.2737  0.000000E+00       3.87241  0.000000E+00       3.87241  0.000000E+00  0.000000E+00      -7.74482       74.5490       2.25418        1       Scatag_D
       8       1660.44       5247.73  0.000000E+00  0.000000E+00       2134.04  0.000000E+00       2983.31       8265.64  0.000000E+00       10606.8       46.9543       10706.3 -1.000000E+10 -1.000000E+10 -1.000000E+10 -1.000000E+10 -1.000000E+10  0.000000E+00  0.000000E+00  0.000000E+00  0.000000E+00  0.000000E+00  0.000000E+00  0.000000E+00  0.000000E+00  0.000000E+00  0.000000E+00  0.000000E+00  0.000000E+00       Funtag_D
       9  4.458513E+05  4.041310E+05       41720.3       22.8463    0.00510563     0.0567847      0.202409      0.227598    0.00311969    0.00131738    0.00768002      0.983424       Opttag_D
       9       73.6723       1.73426       1.00000  0.000000E+00       1.00000  0.000000E+00      0.836445       40.7669       1.00000       44.2394  0.000000E+00       3.86788  0.000000E+00       3.86788  0.000000E+00  0.000000E+00      -7.73576       74.5490       2.25418        1       Scatag_D
       9       1656.16       5270.39  0.000000E+00  0.000000E+00       2133.35  0.000000E+00       2999.56       8281.38  0.000000E+00       10620.9       47.1052       10711.5 -1.000000E+10 -1.000000E+10 -1.000000E+10 -1.000000E+10 -1.000000E+10  0.000000E+00  0.000000E+00  0.000000E+00  0.000000E+00  0.000000E+00  0.000000E+00  0.000000E+00  0.000000E+00  0.000000E+00  0.000000E+00  0.000000E+00  0.000000E+00       Funtag_D
      10  4.458078E+05  4.040652E+05       41742.7       17.6746    0.00560359     0.0648371      0.202338      0.227506    0.00325701    0.00144791    0.00767845      0.983421       Opttag_D
      10       73.7049       1.73710       1.00000  0.000000E+00       1.00000  0.000000E+00      0.836486       40.8152       1.00000       44.2223  0.000000E+00       3.86524  0.000000E+00       3.86524  0.000000E+00  0.000000E+00      -7.73048       74.5490       2.25418        1       Scatag_D
      10       1655.48       5270.36  0.000000E+00  0.000000E+00       2133.05  0.000000E+00       3007.64       8285.54  0.000000E+00       10630.0       47.2278       10713.4 -1.000000E+10 -1.000000E+10 -1.000000E+10 -1.000000E+10 -1.000000E+10  0.000000E+00  0.000000E+00  0.000000E+00  0.000000E+00  0.000000E+00  0.000000E+00  0.000000E+00  0.000000E+00  0.000000E+00  0.000000E+00  0.000000E+00  0.000000E+00       Funtag_D
      11  4.457639E+05  4.039724E+05       41791.5       16.8476    0.00621539     0.0750057      0.202250      0.227405    0.00345045    0.00158024    0.00769154      0.984422       Opttag_D
      11       73.7437       1.74003       1.00000  0.000000E+00       1.00000  0.000000E+00      0.836523       40.8711       1.00000       44.2051  0.000000E+00       3.86193  0.000000E+00       3.86193  0.000000E+00  0.000000E+00      -7.72386       74.5490       2.25418        1       Scatag_D
      11       1661.13       5281.09  0.000000E+00  0.000000E+00       2132.73  0.000000E+00       3015.91       8295.69  0.000000E+00       10641.4       47.4489       10716.1 -1.000000E+10 -1.000000E+10 -1.000000E+10 -1.000000E+10 -1.000000E+10  0.000000E+00  0.000000E+00  0.000000E+00  0.000000E+00  0.000000E+00  0.000000E+00  0.000000E+00  0.000000E+00  0.000000E+00  0.000000E+00  0.000000E+00  0.000000E+00       Funtag_D
      12  4.457179E+05  4.038159E+05       41902.0       21.6567    0.00729030     0.0928086      0.202101      0.227240    0.00377682    0.00177826    0.00772137      0.986770       Opttag_D
      12       73.8079       1.74480       1.00000  0.000000E+00       1.00000  0.000000E+00      0.836583       40.9637       1.00000       44.1749  0.000000E+00       3.85645  0.000000E+00       3.85645  0.000000E+00  0.000000E+00      -7.71291       74.5490       2.25418        1       Scatag_D
      12       1674.04       5306.32  0.000000E+00  0.000000E+00       2132.27  0.000000E+00       3029.56       8326.70  0.000000E+00       10662.0       47.8551       10723.2 -1.000000E+10 -1.000000E+10 -1.000000E+10 -1.000000E+10 -1.000000E+10  0.000000E+00  0.000000E+00  0.000000E+00  0.000000E+00  0.000000E+00  0.000000E+00  0.000000E+00  0.000000E+00  0.000000E+00  0.000000E+00  0.000000E+00  0.000000E+00       Funtag_D
      13  4.456684E+05  4.036420E+05       42026.4       19.0301    0.00861137      0.114708      0.201927      0.227051    0.00413954    0.00199542    0.00774953      0.988366       Opttag_D
      13       73.8822       1.75040       1.00000  0.000000E+00       1.00000  0.000000E+00      0.836654       41.0695       1.00000       44.1382  0.000000E+00       3.85050  0.000000E+00       3.85050  0.000000E+00  0.000000E+00      -7.70099       74.5490       2.25418        1       Scatag_D
      13       1686.27       5323.49  0.000000E+00  0.000000E+00       2131.87  0.000000E+00       3045.07       8368.97  0.000000E+00       10688.5       48.3285       10733.9 -1.000000E+10 -1.000000E+10 -1.000000E+10 -1.000000E+10 -1.000000E+10  0.000000E+00  0.000000E+00  0.000000E+00  0.000000E+00  0.000000E+00  0.000000E+00  0.000000E+00  0.000000E+00  0.000000E+00  0.000000E+00  0.000000E+00  0.000000E+00       Funtag_D
      14  4.456252E+05  4.035443E+05       42080.9       16.7168    0.00946096      0.129609      0.201830      0.226947    0.00434327    0.00210250    0.00776013      0.989568       Opttag_D
      14       73.9249       1.75360       1.00000  0.000000E+00       1.00000  0.000000E+00      0.836692       41.1290       1.00000       44.1145  0.000000E+00       3.84752  0.000000E+00       3.84752  0.000000E+00  0.000000E+00      -7.69504       74.5490       2.25418        1       Scatag_D
      14       1690.89       5336.45  0.000000E+00  0.000000E+00       2131.73  0.000000E+00       3053.05       8374.33  0.000000E+00       10705.7       48.5620       10740.2 -1.000000E+10 -1.000000E+10 -1.000000E+10 -1.000000E+10 -1.000000E+10  0.000000E+00  0.000000E+00  0.000000E+00  0.000000E+00  0.000000E+00  0.000000E+00  0.000000E+00  0.000000E+00  0.000000E+00  0.000000E+00  0.000000E+00  0.000000E+00       Funtag_D
      15  4.455832E+05  4.034485E+05       42134.7       15.5094     0.0104537      0.147610      0.201728      0.226834    0.00454311    0.00222018    0.00776224      0.990938       Opttag_D
      15       73.9703       1.75715       1.00000  0.000000E+00       1.00000  0.000000E+00      0.836734       41.1909       1.00000       44.0860  0.000000E+00       3.84487  0.000000E+00       3.84487  0.000000E+00  0.000000E+00      -7.68973       74.5490       2.25418        1       Scatag_D
      15       1691.81       5351.24  0.000000E+00  0.000000E+00       2131.65  0.000000E+00       3060.72       8376.95  0.000000E+00       10725.9       48.7576       10747.7 -1.000000E+10 -1.000000E+10 -1.000000E+10 -1.000000E+10 -1.000000E+10  0.000000E+00  0.000000E+00  0.000000E+00  0.000000E+00  0.000000E+00  0.000000E+00  0.000000E+00  0.000000E+00  0.000000E+00  0.000000E+00  0.000000E+00  0.000000E+00       Funtag_D
      16  4.455571E+05  4.033688E+05       42188.3       18.0192     0.0113888      0.165096      0.201640      0.226743    0.00470909    0.00230196    0.00776443      0.992263       Opttag_D
      16       74.0091       1.76015       1.00000  0.000000E+00       1.00000  0.000000E+00      0.836765       41.2423       1.00000       44.0610  0.000000E+00       3.84276  0.000000E+00       3.84276  0.000000E+00  0.000000E+00      -7.68552       74.5490       2.25418        1       Scatag_D
      16       1692.76       5365.56  0.000000E+00  0.000000E+00       2131.70  0.000000E+00       3066.16       8383.41  0.000000E+00       10745.0       48.8898       10754.7 -1.000000E+10 -1.000000E+10 -1.000000E+10 -1.000000E+10 -1.000000E+10  0.000000E+00  0.000000E+00  0.000000E+00  0.000000E+00  0.000000E+00  0.000000E+00  0.000000E+00  0.000000E+00  0.000000E+00  0.000000E+00  0.000000E+00  0.000000E+00       Funtag_D
      17  4.455274E+05  4.033363E+05       42191.1       14.2648     0.0118512      0.174512      0.201603      0.226708    0.00477690    0.00233707    0.00776154      0.992405       Opttag_D
      17       74.0247       1.76125       1.00000  0.000000E+00       1.00000  0.000000E+00      0.836771       41.2609       1.00000       44.0504  0.000000E+00       3.84217  0.000000E+00       3.84217  0.000000E+00  0.000000E+00      -7.68435       74.5490       2.25418        1       Scatag_D
      17       1691.51       5367.09  0.000000E+00  0.000000E+00       2131.74  0.000000E+00       3066.83       8374.02  0.000000E+00       10753.9       48.8910       10757.2 -1.000000E+10 -1.000000E+10 -1.000000E+10 -1.000000E+10 -1.000000E+10  0.000000E+00  0.000000E+00  0.000000E+00  0.000000E+00  0.000000E+00  0.000000E+00  0.000000E+00  0.000000E+00  0.000000E+00  0.000000E+00  0.000000E+00  0.000000E+00       Funtag_D
      18  4.454845E+05  4.032587E+05       42225.7       14.2119     0.0129153      0.195938      0.201524      0.226631    0.00493869    0.00241766    0.00776636      0.993663       Opttag_D
      18       74.0599       1.76356       1.00000  0.000000E+00       1.00000  0.000000E+00      0.836775       41.3017       1.00000       44.0308  0.000000E+00       3.84046  0.000000E+00       3.84046  0.000000E+00  0.000000E+00      -7.68093       74.5490       2.25418        1       Scatag_D
      18       1693.60       5380.70  0.000000E+00  0.000000E+00       2131.86  0.000000E+00       3067.96       8365.40  0.000000E+00       10773.7       48.9229       10763.5 -1.000000E+10 -1.000000E+10 -1.000000E+10 -1.000000E+10 -1.000000E+10  0.000000E+00  0.000000E+00  0.000000E+00  0.000000E+00  0.000000E+00  0.000000E+00  0.000000E+00  0.000000E+00  0.000000E+00  0.000000E+00  0.000000E+00  0.000000E+00       Funtag_D
      19  4.454586E+05  4.031595E+05       42299.1       21.1795     0.0143326      0.223673      0.201422      0.226526    0.00514562    0.00252133    0.00778509      0.995926       Opttag_D
      19       74.1059       1.76655       1.00000  0.000000E+00       1.00000  0.000000E+00      0.836775       41.3547       1.00000       44.0087  0.000000E+00       3.83796  0.000000E+00       3.83796  0.000000E+00  0.000000E+00      -7.67592       74.5490       2.25418        1       Scatag_D
      19       1701.78       5405.24  0.000000E+00  0.000000E+00       2132.13  0.000000E+00       3069.87       8368.15  0.000000E+00       10799.5       49.0361       10773.4 -1.000000E+10 -1.000000E+10 -1.000000E+10 -1.000000E+10 -1.000000E+10  0.000000E+00  0.000000E+00  0.000000E+00  0.000000E+00  0.000000E+00  0.000000E+00  0.000000E+00  0.000000E+00  0.000000E+00  0.000000E+00  0.000000E+00  0.000000E+00       Funtag_D
      20  4.454278E+05  4.031248E+05       42303.0       14.1233     0.0148935      0.234741      0.201387      0.226488    0.00521802    0.00256434    0.00778726      0.995325       Opttag_D
      20       74.1225       1.76760       1.00000  0.000000E+00       1.00000  0.000000E+00      0.836769       41.3725       1.00000       44.0018  0.000000E+00       3.83706  0.000000E+00       3.83706  0.000000E+00  0.000000E+00      -7.67412       74.5490       2.25418        1       Scatag_D
      20       1702.73       5398.72  0.000000E+00  0.000000E+00       2132.23  0.000000E+00       3070.19       8364.74  0.000000E+00       10809.2       49.0787       10776.1 -1.000000E+10 -1.000000E+10 -1.000000E+10 -1.000000E+10 -1.000000E+10  0.000000E+00  0.000000E+00  0.000000E+00  0.000000E+00  0.000000E+00  0.000000E+00  0.000000E+00  0.000000E+00  0.000000E+00  0.000000E+00  0.000000E+00  0.000000E+00       Funtag_D
      21  4.453948E+05  4.030745E+05       42320.2       12.3980     0.0158508      0.253576      0.201333      0.226426    0.00532278    0.00263817    0.00779377      0.995696       Opttag_D
      21       74.1489       1.76932       1.00000  0.000000E+00       1.00000  0.000000E+00      0.836757       41.3995       1.00000       43.9909  0.000000E+00       3.83574  0.000000E+00       3.83574  0.000000E+00  0.000000E+00      -7.67148       74.5490       2.25418        1       Scatag_D
      21       1705.58       5402.74  0.000000E+00  0.000000E+00       2132.46  0.000000E+00       3070.43       8355.28  0.000000E+00       10824.8       49.1692       10779.8 -1.000000E+10 -1.000000E+10 -1.000000E+10 -1.000000E+10 -1.000000E+10  0.000000E+00  0.000000E+00  0.000000E+00  0.000000E+00  0.000000E+00  0.000000E+00  0.000000E+00  0.000000E+00  0.000000E+00  0.000000E+00  0.000000E+00  0.000000E+00       Funtag_D
      22  4.453679E+05  4.030353E+05       42332.6       13.4856     0.0169303      0.274473      0.201281      0.226361    0.00540467    0.00271809    0.00779197      0.996393       Opttag_D
      22       74.1758       1.77131       1.00000  0.000000E+00       1.00000  0.000000E+00      0.836748       41.4255       1.00000       43.9766  0.000000E+00       3.83482  0.000000E+00       3.83482  0.000000E+00  0.000000E+00      -7.66964       74.5490       2.25418        1       Scatag_D
      22       1704.80       5410.31  0.000000E+00  0.000000E+00       2132.81  0.000000E+00       3070.96       8338.86  0.000000E+00       10841.4       49.2589       10784.2 -1.000000E+10 -1.000000E+10 -1.000000E+10 -1.000000E+10 -1.000000E+10  0.000000E+00  0.000000E+00  0.000000E+00  0.000000E+00  0.000000E+00  0.000000E+00  0.000000E+00  0.000000E+00  0.000000E+00  0.000000E+00  0.000000E+00  0.000000E+00       Funtag_D
      23  4.453371E+05  4.029744E+05       42362.8       19.8133     0.0194188      0.321358      0.201187      0.226236    0.00553171    0.00287680    0.00778018      0.998389       Opttag_D
      23       74.2314       1.77579       1.00000  0.000000E+00       1.00000  0.000000E+00      0.836731       41.4768       1.00000       43.9429  0.000000E+00       3.83339  0.000000E+00       3.83339  0.000000E+00  0.000000E+00      -7.66678       74.5490       2.25418        1       Scatag_D
      23       1699.64       5432.01  0.000000E+00  0.000000E+00       2133.82  0.000000E+00       3072.63       8302.10  0.000000E+00       10877.6       49.4610       10795.5 -1.000000E+10 -1.000000E+10 -1.000000E+10 -1.000000E+10 -1.000000E+10  0.000000E+00  0.000000E+00  0.000000E+00  0.000000E+00  0.000000E+00  0.000000E+00  0.000000E+00  0.000000E+00  0.000000E+00  0.000000E+00  0.000000E+00  0.000000E+00       Funtag_D
      24  4.453027E+05  4.029718E+05       42330.8       12.3062     0.0200969      0.333783      0.201175      0.226205    0.00553700    0.00293035    0.00775924      0.997751       Opttag_D
      24       74.2423       1.77679       1.00000  0.000000E+00       1.00000  0.000000E+00      0.836726       41.4841       1.00000       43.9328  0.000000E+00       3.83345  0.000000E+00       3.83345  0.000000E+00  0.000000E+00      -7.66690       74.5490       2.25418        1       Scatag_D
      24       1690.50       5425.07  0.000000E+00  0.000000E+00       2134.19  0.000000E+00       3073.28       8275.80  0.000000E+00       10885.5       49.4877       10797.0 -1.000000E+10 -1.000000E+10 -1.000000E+10 -1.000000E+10 -1.000000E+10  0.000000E+00  0.000000E+00  0.000000E+00  0.000000E+00  0.000000E+00  0.000000E+00  0.000000E+00  0.000000E+00  0.000000E+00  0.000000E+00  0.000000E+00  0.000000E+00       Funtag_D
      25  4.452808E+05  4.029778E+05       42303.0       10.9291     0.0203760      0.338671      0.201178      0.226200    0.00552462    0.00295302    0.00774533      0.997670       Opttag_D
      25       74.2435       1.77694       1.00000  0.000000E+00       1.00000  0.000000E+00      0.836722       41.4826       1.00000       43.9280  0.000000E+00       3.83375  0.000000E+00       3.83375  0.000000E+00  0.000000E+00      -7.66749       74.5490       2.25418        1       Scatag_D
      25       1684.45       5424.19  0.000000E+00  0.000000E+00       2134.42  0.000000E+00       3073.81       8253.78  0.000000E+00       10887.0       49.4753       10795.9 -1.000000E+10 -1.000000E+10 -1.000000E+10 -1.000000E+10 -1.000000E+10  0.000000E+00  0.000000E+00  0.000000E+00  0.000000E+00  0.000000E+00  0.000000E+00  0.000000E+00  0.000000E+00  0.000000E+00  0.000000E+00  0.000000E+00  0.000000E+00       Funtag_D
      26  4.452489E+05  4.029657E+05       42283.2       12.4699     0.0213858      0.356134      0.201159      0.226167    0.00554978    0.00302888    0.00772906      0.997894       Opttag_D
      26       74.2555       1.77777       1.00000  0.000000E+00       1.00000  0.000000E+00      0.836705       41.4906       1.00000       43.9150  0.000000E+00       3.83366  0.000000E+00       3.83366  0.000000E+00  0.000000E+00      -7.66731       74.5490       2.25418        1       Scatag_D
      26       1677.38       5426.63  0.000000E+00  0.000000E+00       2135.11  0.000000E+00       3075.84       8225.19  0.000000E+00       10896.7       49.4961       10796.8 -1.000000E+10 -1.000000E+10 -1.000000E+10 -1.000000E+10 -1.000000E+10  0.000000E+00  0.000000E+00  0.000000E+00  0.000000E+00  0.000000E+00  0.000000E+00  0.000000E+00  0.000000E+00  0.000000E+00  0.000000E+00  0.000000E+00  0.000000E+00       Funtag_D
      27  4.452268E+05  4.029048E+05       42322.1       21.8269     0.0235361      0.391959      0.201100      0.226096    0.00567682    0.00317371    0.00772731      0.999403       Opttag_D
      27       74.2847       1.77926       1.00000  0.000000E+00       1.00000  0.000000E+00      0.836657       41.5171       1.00000       43.8932  0.000000E+00       3.83240  0.000000E+00       3.83240  0.000000E+00  0.000000E+00      -7.66480       74.5490       2.25418        1       Scatag_D
      27       1676.62       5443.05  0.000000E+00  0.000000E+00       2136.75  0.000000E+00       3080.98       8214.60  0.000000E+00       10918.4       49.5415       10802.1 -1.000000E+10 -1.000000E+10 -1.000000E+10 -1.000000E+10 -1.000000E+10  0.000000E+00  0.000000E+00  0.000000E+00  0.000000E+00  0.000000E+00  0.000000E+00  0.000000E+00  0.000000E+00  0.000000E+00  0.000000E+00  0.000000E+00  0.000000E+00       Funtag_D
      28  4.451887E+05  4.028732E+05       42315.5       11.6142     0.0242745      0.403982      0.201073      0.226069    0.00574273    0.00322235    0.00772966      0.998465       Opttag_D
      28       74.2960       1.77967       1.00000  0.000000E+00       1.00000  0.000000E+00      0.836638       41.5289       1.00000       43.8878  0.000000E+00       3.83173  0.000000E+00       3.83173  0.000000E+00  0.000000E+00      -7.66347       74.5490       2.25418        1       Scatag_D
      28       1677.64       5432.84  0.000000E+00  0.000000E+00       2137.29  0.000000E+00       3082.77       8204.61  0.000000E+00       10926.6       49.5557       10804.2 -1.000000E+10 -1.000000E+10 -1.000000E+10 -1.000000E+10 -1.000000E+10  0.000000E+00  0.000000E+00  0.000000E+00  0.000000E+00  0.000000E+00  0.000000E+00  0.000000E+00  0.000000E+00  0.000000E+00  0.000000E+00  0.000000E+00  0.000000E+00       Funtag_D
      29  4.451634E+05  4.028204E+05       42343.0       9.94007     0.0252381      0.419378      0.201033      0.226035    0.00585278    0.00327607    0.00774536      0.998532       Opttag_D
      29       74.3113       1.78008       1.00000  0.000000E+00       1.00000  0.000000E+00      0.836610       41.5473       1.00000       43.8821  0.000000E+00       3.83062  0.000000E+00       3.83062  0.000000E+00  0.000000E+00      -7.66125       74.5490       2.25418        1       Scatag_D
      29       1684.46       5433.57  0.000000E+00  0.000000E+00       2138.05  0.000000E+00       3085.21       8208.53  0.000000E+00       10937.1       49.5667       10806.5 -1.000000E+10 -1.000000E+10 -1.000000E+10 -1.000000E+10 -1.000000E+10  0.000000E+00  0.000000E+00  0.000000E+00  0.000000E+00  0.000000E+00  0.000000E+00  0.000000E+00  0.000000E+00  0.000000E+00  0.000000E+00  0.000000E+00  0.000000E+00       Funtag_D
      30  4.451383E+05  4.027492E+05       42389.1       11.5280     0.0266912      0.441925      0.200980      0.225990    0.00600129    0.00334259    0.00776479      0.998867       Opttag_D
      30       74.3319       1.78065       1.00000  0.000000E+00       1.00000  0.000000E+00      0.836572       41.5724       1.00000       43.8717  0.000000E+00       3.82921  0.000000E+00       3.82921  0.000000E+00  0.000000E+00      -7.65843       74.5490       2.25418        1       Scatag_D
      30       1692.92       5437.22  0.000000E+00  0.000000E+00       2139.33  0.000000E+00       3089.18       8219.96  0.000000E+00       10951.5       49.5721       10809.4 -1.000000E+10 -1.000000E+10 -1.000000E+10 -1.000000E+10 -1.000000E+10  0.000000E+00  0.000000E+00  0.000000E+00  0.000000E+00  0.000000E+00  0.000000E+00  0.000000E+00  0.000000E+00  0.000000E+00  0.000000E+00  0.000000E+00  0.000000E+00       Funtag_D
      31  4.451155E+05  4.026333E+05       42482.2       21.3166     0.0294151      0.481998      0.200889      0.225930    0.00624297    0.00339189    0.00779952       1.00028       Opttag_D
      31       74.3656       1.78171       1.00000  0.000000E+00       1.00000  0.000000E+00      0.836511       41.6172       1.00000       43.8505  0.000000E+00       3.82726  0.000000E+00       3.82726  0.000000E+00  0.000000E+00      -7.65452       74.5490       2.25418        1       Scatag_D
      31       1708.10       5452.62  0.000000E+00  0.000000E+00       2142.00  0.000000E+00       3096.90       8244.67  0.000000E+00       10975.1       49.5827       10813.2 -1.000000E+10 -1.000000E+10 -1.000000E+10 -1.000000E+10 -1.000000E+10  0.000000E+00  0.000000E+00  0.000000E+00  0.000000E+00  0.000000E+00  0.000000E+00  0.000000E+00  0.000000E+00  0.000000E+00  0.000000E+00  0.000000E+00  0.000000E+00       Funtag_D
      32  4.450783E+05  4.025933E+05       42484.9       12.4373     0.0306197      0.498896      0.200854      0.225901    0.00632636    0.00342060    0.00779782      0.999483       Opttag_D
      32       74.3781       1.78223       1.00000  0.000000E+00       1.00000  0.000000E+00      0.836489       41.6344       1.00000       43.8388  0.000000E+00       3.82643  0.000000E+00       3.82643  0.000000E+00  0.000000E+00      -7.65286       74.5490       2.25418        1       Scatag_D
      32       1707.35       5443.93  0.000000E+00  0.000000E+00       2143.33  0.000000E+00       3100.46       8241.80  0.000000E+00       10984.3       49.5437       10814.2 -1.000000E+10 -1.000000E+10 -1.000000E+10 -1.000000E+10 -1.000000E+10  0.000000E+00  0.000000E+00  0.000000E+00  0.000000E+00  0.000000E+00  0.000000E+00  0.000000E+00  0.000000E+00  0.000000E+00  0.000000E+00  0.000000E+00  0.000000E+00       Funtag_D
      33  4.450518E+05  4.025770E+05       42474.8       10.7693     0.0314668      0.509505      0.200840      0.225889    0.00636021    0.00342162    0.00779002      0.999495       Opttag_D
      33       74.3831       1.78251       1.00000  0.000000E+00       1.00000  0.000000E+00      0.836483       41.6433       1.00000       43.8278  0.000000E+00       3.82628  0.000000E+00       3.82628  0.000000E+00  0.000000E+00      -7.65256       74.5490       2.25418        1       Scatag_D
      33       1703.94       5444.05  0.000000E+00  0.000000E+00       2144.42  0.000000E+00       3102.78       8229.60  0.000000E+00       10988.0       49.4902       10812.5 -1.000000E+10 -1.000000E+10 -1.000000E+10 -1.000000E+10 -1.000000E+10  0.000000E+00  0.000000E+00  0.000000E+00  0.000000E+00  0.000000E+00  0.000000E+00  0.000000E+00  0.000000E+00  0.000000E+00  0.000000E+00  0.000000E+00  0.000000E+00       Funtag_D
      34  4.450206E+05  4.025477E+05       42472.9       13.0682     0.0331034      0.529381      0.200813      0.225867    0.00642153    0.00340481    0.00778088      0.999703       Opttag_D
      34       74.3927       1.78306       1.00000  0.000000E+00       1.00000  0.000000E+00      0.836468       41.6603       1.00000       43.8092  0.000000E+00       3.82592  0.000000E+00       3.82592  0.000000E+00  0.000000E+00      -7.65183       74.5490       2.25418        1       Scatag_D
      34       1699.94       5446.32  0.000000E+00  0.000000E+00       2146.43  0.000000E+00       3106.73       8218.91  0.000000E+00       10995.1       49.4224       10810.1 -1.000000E+10 -1.000000E+10 -1.000000E+10 -1.000000E+10 -1.000000E+10  0.000000E+00  0.000000E+00  0.000000E+00  0.000000E+00  0.000000E+00  0.000000E+00  0.000000E+00  0.000000E+00  0.000000E+00  0.000000E+00  0.000000E+00  0.000000E+00       Funtag_D
      35  4.449968E+05  4.025017E+05       42495.1       20.6363     0.0359297      0.562119      0.200776      0.225818    0.00651731    0.00345338    0.00777792       1.00028       Opttag_D
      35       74.4079       1.78388       1.00000  0.000000E+00       1.00000  0.000000E+00      0.836442       41.6862       1.00000       43.7830  0.000000E+00       3.82532  0.000000E+00       3.82532  0.000000E+00  0.000000E+00      -7.65064       74.5490       2.25418        1       Scatag_D
      35       1698.65       5452.61  0.000000E+00  0.000000E+00       2150.25  0.000000E+00       3112.50       8217.09  0.000000E+00       11007.2       49.2933       10807.4 -1.000000E+10 -1.000000E+10 -1.000000E+10 -1.000000E+10 -1.000000E+10  0.000000E+00  0.000000E+00  0.000000E+00  0.000000E+00  0.000000E+00  0.000000E+00  0.000000E+00  0.000000E+00  0.000000E+00  0.000000E+00  0.000000E+00  0.000000E+00       Funtag_D
      36  4.449620E+05  4.024873E+05       42474.7       12.3116     0.0365971      0.569230      0.200764      0.225809    0.00654737    0.00343687    0.00777396      0.999583       Opttag_D
      36       74.4102       1.78376       1.00000  0.000000E+00       1.00000  0.000000E+00      0.836430       41.6898       1.00000       43.7805  0.000000E+00       3.82513  0.000000E+00       3.82513  0.000000E+00  0.000000E+00      -7.65026       74.5490       2.25418        1       Scatag_D
      36       1696.92       5445.02  0.000000E+00  0.000000E+00       2151.04  0.000000E+00       3112.89       8204.82  0.000000E+00       11009.0       49.2612       10805.7 -1.000000E+10 -1.000000E+10 -1.000000E+10 -1.000000E+10 -1.000000E+10  0.000000E+00  0.000000E+00  0.000000E+00  0.000000E+00  0.000000E+00  0.000000E+00  0.000000E+00  0.000000E+00  0.000000E+00  0.000000E+00  0.000000E+00  0.000000E+00       Funtag_D
      37  4.449349E+05  4.024644E+05       42470.5       10.4940     0.0375311      0.578915      0.200753      0.225791    0.00659530    0.00345338    0.00777832      0.999469       Opttag_D
      37       74.4129       1.78340       1.00000  0.000000E+00       1.00000  0.000000E+00      0.836410       41.6932       1.00000       43.7796  0.000000E+00       3.82485  0.000000E+00       3.82485  0.000000E+00  0.000000E+00      -7.64970       74.5490       2.25418        1       Scatag_D
      37       1698.83       5443.77  0.000000E+00  0.000000E+00       2152.17  0.000000E+00       3112.68       8200.01  0.000000E+00       11011.1       49.2222       10802.7 -1.000000E+10 -1.000000E+10 -1.000000E+10 -1.000000E+10 -1.000000E+10  0.000000E+00  0.000000E+00  0.000000E+00  0.000000E+00  0.000000E+00  0.000000E+00  0.000000E+00  0.000000E+00  0.000000E+00  0.000000E+00  0.000000E+00  0.000000E+00       Funtag_D
      38  4.449102E+05  4.024321E+05       42478.2       11.3765     0.0389172      0.593394      0.200739      0.225769    0.00666262    0.00348485    0.00778540      0.999513       Opttag_D
      38       74.4163       1.78286       1.00000  0.000000E+00       1.00000  0.000000E+00      0.836382       41.6978       1.00000       43.7776  0.000000E+00       3.82454  0.000000E+00       3.82454  0.000000E+00  0.000000E+00      -7.64909       74.5490       2.25418        1       Scatag_D
      38       1701.92       5444.24  0.000000E+00  0.000000E+00       2154.01  0.000000E+00       3112.87       8202.21  0.000000E+00       11014.4       49.1731       10799.3 -1.000000E+10 -1.000000E+10 -1.000000E+10 -1.000000E+10 -1.000000E+10  0.000000E+00  0.000000E+00  0.000000E+00  0.000000E+00  0.000000E+00  0.000000E+00  0.000000E+00  0.000000E+00  0.000000E+00  0.000000E+00  0.000000E+00  0.000000E+00       Funtag_D
      39  4.448854E+05  4.023734E+05       42512.1       21.0969     0.0419547      0.624544      0.200711      0.225727    0.00678505    0.00354874    0.00780112       1.00025       Opttag_D
      39       74.4179       1.78113       1.00000  0.000000E+00       1.00000  0.000000E+00      0.836328       41.6971       1.00000       43.7752  0.000000E+00       3.82474  0.000000E+00       3.82474  0.000000E+00  0.000000E+00      -7.64948       74.5490       2.25418        1       Scatag_D
      39       1708.80       5452.26  0.000000E+00  0.000000E+00       2158.21  0.000000E+00       3112.67       8218.30  0.000000E+00       11020.3       49.1638       10792.4 -1.000000E+10 -1.000000E+10 -1.000000E+10 -1.000000E+10 -1.000000E+10  0.000000E+00  0.000000E+00  0.000000E+00  0.000000E+00  0.000000E+00  0.000000E+00  0.000000E+00  0.000000E+00  0.000000E+00  0.000000E+00  0.000000E+00  0.000000E+00       Funtag_D
      40  4.448473E+05  4.023502E+05       42497.1       11.7194     0.0434042      0.639358      0.200700      0.225714    0.00683329    0.00355341    0.00779979      0.999764       Opttag_D
      40       74.4187       1.78052       1.00000  0.000000E+00       1.00000  0.000000E+00      0.836303       41.6993       1.00000       43.7725  0.000000E+00       3.82482  0.000000E+00       3.82482  0.000000E+00  0.000000E+00      -7.64964       74.5490       2.25418        1       Scatag_D
      40       1708.22       5446.99  0.000000E+00  0.000000E+00       2160.36  0.000000E+00       3113.07       8206.00  0.000000E+00       11023.5       49.1849       10789.7 -1.000000E+10 -1.000000E+10 -1.000000E+10 -1.000000E+10 -1.000000E+10  0.000000E+00  0.000000E+00  0.000000E+00  0.000000E+00  0.000000E+00  0.000000E+00  0.000000E+00  0.000000E+00  0.000000E+00  0.000000E+00  0.000000E+00  0.000000E+00       Funtag_D
      41  4.448271E+05  4.023451E+05       42482.0       9.80930     0.0440183      0.645729      0.200699      0.225714    0.00684388    0.00354360    0.00779378      0.999739       Opttag_D
      41       74.4160       1.78003       1.00000  0.000000E+00       1.00000  0.000000E+00      0.836294       41.6968       1.00000       43.7704  0.000000E+00       3.82525  0.000000E+00       3.82525  0.000000E+00  0.000000E+00      -7.65050       74.5490       2.25418        1       Scatag_D
      41       1705.59       5446.71  0.000000E+00  0.000000E+00       2161.24  0.000000E+00       3112.78       8195.64  0.000000E+00       11023.7       49.2360       10787.1 -1.000000E+10 -1.000000E+10 -1.000000E+10 -1.000000E+10 -1.000000E+10  0.000000E+00  0.000000E+00  0.000000E+00  0.000000E+00  0.000000E+00  0.000000E+00  0.000000E+00  0.000000E+00  0.000000E+00  0.000000E+00  0.000000E+00  0.000000E+00       Funtag_D
      42  4.448007E+05  4.023339E+05       42466.8       10.8720     0.0454601      0.660404      0.200700      0.225714    0.00686735    0.00351309    0.00778562      0.999936       Opttag_D
      42       74.4102       1.77894       1.00000  0.000000E+00       1.00000  0.000000E+00      0.836273       41.6914       1.00000       43.7663  0.000000E+00       3.82613  0.000000E+00       3.82613  0.000000E+00  0.000000E+00      -7.65227       74.5490       2.25418        1       Scatag_D
      42       1702.02       5448.86  0.000000E+00  0.000000E+00       2163.27  0.000000E+00       3112.23       8184.40  0.000000E+00       11024.4       49.3424       10782.2 -1.000000E+10 -1.000000E+10 -1.000000E+10 -1.000000E+10 -1.000000E+10  0.000000E+00  0.000000E+00  0.000000E+00  0.000000E+00  0.000000E+00  0.000000E+00  0.000000E+00  0.000000E+00  0.000000E+00  0.000000E+00  0.000000E+00  0.000000E+00       Funtag_D
      43  4.447823E+05  4.023093E+05       42473.0       18.9826     0.0480750      0.686380      0.200696      0.225709    0.00691848    0.00343430    0.00777985       1.00037       Opttag_D
      43       74.4011       1.77711       1.00000  0.000000E+00       1.00000  0.000000E+00      0.836230       41.6856       1.00000       43.7613  0.000000E+00       3.82715  0.000000E+00       3.82715  0.000000E+00  0.000000E+00      -7.65430       74.5490       2.25418        1       Scatag_D
      43       1699.49       5453.61  0.000000E+00  0.000000E+00       2166.89  0.000000E+00       3111.49       8190.61  0.000000E+00       11026.0       49.4863       10775.4 -1.000000E+10 -1.000000E+10 -1.000000E+10 -1.000000E+10 -1.000000E+10  0.000000E+00  0.000000E+00  0.000000E+00  0.000000E+00  0.000000E+00  0.000000E+00  0.000000E+00  0.000000E+00  0.000000E+00  0.000000E+00  0.000000E+00  0.000000E+00       Funtag_D
      44  4.447518E+05  4.022997E+05       42452.1       11.2304     0.0489536      0.694894      0.200695      0.225711    0.00693855    0.00340702    0.00777888       1.00019       Opttag_D
      44       74.3976       1.77639       1.00000  0.000000E+00       1.00000  0.000000E+00      0.836215       41.6825       1.00000       43.7606  0.000000E+00       3.82752  0.000000E+00       3.82752  0.000000E+00  0.000000E+00      -7.65505       74.5490       2.25418        1       Scatag_D
      44       1699.07       5451.63  0.000000E+00  0.000000E+00       2168.13  0.000000E+00       3111.26       8172.87  0.000000E+00       11026.4       49.5097       10773.2 -1.000000E+10 -1.000000E+10 -1.000000E+10 -1.000000E+10 -1.000000E+10  0.000000E+00  0.000000E+00  0.000000E+00  0.000000E+00  0.000000E+00  0.000000E+00  0.000000E+00  0.000000E+00  0.000000E+00  0.000000E+00  0.000000E+00  0.000000E+00       Funtag_D
      45  4.447317E+05  4.022844E+05       42447.2       9.97069     0.0498420      0.703288      0.200689      0.225711    0.00697051    0.00338184    0.00778152       1.00013       Opttag_D
      45       74.3941       1.77556       1.00000  0.000000E+00       1.00000  0.000000E+00      0.836194       41.6802       1.00000       43.7611  0.000000E+00       3.82765  0.000000E+00       3.82765  0.000000E+00  0.000000E+00      -7.65529       74.5490       2.25418        1       Scatag_D
      45       1700.22       5451.01  0.000000E+00  0.000000E+00       2169.33  0.000000E+00       3110.94       8169.32  0.000000E+00       11026.2       49.5045       10770.7 -1.000000E+10 -1.000000E+10 -1.000000E+10 -1.000000E+10 -1.000000E+10  0.000000E+00  0.000000E+00  0.000000E+00  0.000000E+00  0.000000E+00  0.000000E+00  0.000000E+00  0.000000E+00  0.000000E+00  0.000000E+00  0.000000E+00  0.000000E+00       Funtag_D
      46  4.447108E+05  4.022600E+05       42450.8       11.0836     0.0512332      0.716225      0.200689      0.225713    0.00702144    0.00334480    0.00778715       1.00019       Opttag_D
      46       74.3885       1.77420       1.00000  0.000000E+00       1.00000  0.000000E+00      0.836162       41.6763       1.00000       43.7625  0.000000E+00       3.82784  0.000000E+00       3.82784  0.000000E+00  0.000000E+00      -7.65568       74.5490       2.25418        1       Scatag_D
      46       1702.69       5451.62  0.000000E+00  0.000000E+00       2171.24  0.000000E+00       3110.58       8172.33  0.000000E+00       11025.8       49.5118       10767.0 -1.000000E+10 -1.000000E+10 -1.000000E+10 -1.000000E+10 -1.000000E+10  0.000000E+00  0.000000E+00  0.000000E+00  0.000000E+00  0.000000E+00  0.000000E+00  0.000000E+00  0.000000E+00  0.000000E+00  0.000000E+00  0.000000E+00  0.000000E+00       Funtag_D
      47  4.446782E+05  4.022180E+05       42460.2       15.4409     0.0539935      0.768949      0.200687      0.225725    0.00710893    0.00329695    0.00779340       1.00027       Opttag_D
      47       74.3748       1.77122       1.00000  0.000000E+00       1.00000  0.000000E+00      0.836098       41.6624       1.00000       43.7668  0.000000E+00       3.82868  0.000000E+00       3.82868  0.000000E+00  0.000000E+00      -7.65737       74.5490       2.25418        1       Scatag_D
      47       1705.42       5452.45  0.000000E+00  0.000000E+00       2175.13  0.000000E+00       3110.24       8183.46  0.000000E+00       11024.4       49.5621       10759.6 -1.000000E+10 -1.000000E+10 -1.000000E+10 -1.000000E+10 -1.000000E+10  0.000000E+00  0.000000E+00  0.000000E+00  0.000000E+00  0.000000E+00  0.000000E+00  0.000000E+00  0.000000E+00  0.000000E+00  0.000000E+00  0.000000E+00  0.000000E+00       Funtag_D
      48  4.446432E+05  4.021799E+05       42463.3       12.6203     0.0574300      0.837900      0.200695      0.225755    0.00718852    0.00319321    0.00779784       1.00034       Opttag_D
      48       74.3572       1.76774       1.00000  0.000000E+00       1.00000  0.000000E+00      0.836024       41.6443       1.00000       43.7733  0.000000E+00       3.82996  0.000000E+00       3.82996  0.000000E+00  0.000000E+00      -7.65993       74.5490       2.25418        1       Scatag_D
      48       1707.36       5453.28  0.000000E+00  0.000000E+00       2180.02  0.000000E+00       3110.41       8186.35  0.000000E+00       11023.5       49.6914       10752.7 -1.000000E+10 -1.000000E+10 -1.000000E+10 -1.000000E+10 -1.000000E+10  0.000000E+00  0.000000E+00  0.000000E+00  0.000000E+00  0.000000E+00  0.000000E+00  0.000000E+00  0.000000E+00  0.000000E+00  0.000000E+00  0.000000E+00  0.000000E+00       Funtag_D
      49  4.446168E+05  4.021779E+05       42438.9       9.78228     0.0583456      0.856901      0.200704      0.225767    0.00719262    0.00317485    0.00778918       1.00020       Opttag_D
      49       74.3497       1.76660       1.00000  0.000000E+00       1.00000  0.000000E+00      0.836005       41.6340       1.00000       43.7759  0.000000E+00       3.83080  0.000000E+00       3.83080  0.000000E+00  0.000000E+00      -7.66160       74.5490       2.25418        1       Scatag_D
      49       1703.57       5451.77  0.000000E+00  0.000000E+00       2181.28  0.000000E+00       3110.33       8171.96  0.000000E+00       11021.8       49.7542       10748.4 -1.000000E+10 -1.000000E+10 -1.000000E+10 -1.000000E+10 -1.000000E+10  0.000000E+00  0.000000E+00  0.000000E+00  0.000000E+00  0.000000E+00  0.000000E+00  0.000000E+00  0.000000E+00  0.000000E+00  0.000000E+00  0.000000E+00  0.000000E+00       Funtag_D
      50  4.445822E+05  4.021847E+05       42397.4       11.0388     0.0601322      0.894115      0.200731      0.225788    0.00717834    0.00316198    0.00776831       1.00001       Opttag_D
      50       74.3335       1.76431       1.00000  0.000000E+00       1.00000  0.000000E+00      0.835972       41.6095       1.00000       43.7812  0.000000E+00       3.83266  0.000000E+00       3.83266  0.000000E+00  0.000000E+00      -7.66532       74.5490       2.25418        1       Scatag_D
      50       1694.46       5449.65  0.000000E+00  0.000000E+00       2183.75  0.000000E+00       3110.25       8152.49  0.000000E+00       11017.9       49.8632       10739.1 -1.000000E+10 -1.000000E+10 -1.000000E+10 -1.000000E+10 -1.000000E+10  0.000000E+00  0.000000E+00  0.000000E+00  0.000000E+00  0.000000E+00  0.000000E+00  0.000000E+00  0.000000E+00  0.000000E+00  0.000000E+00  0.000000E+00  0.000000E+00       Funtag_D
      51  4.445608E+05  4.021874E+05       42373.5       14.7351     0.0623964      0.939994      0.200756      0.225816    0.00717284    0.00311544    0.00775291      0.999881       Opttag_D
      51       74.3163       1.76179       1.00000  0.000000E+00       1.00000  0.000000E+00      0.835929       41.5844       1.00000       43.7904  0.000000E+00       3.83449  0.000000E+00       3.83449  0.000000E+00  0.000000E+00      -7.66899       74.5490       2.25418        1       Scatag_D
      51       1687.75       5448.26  0.000000E+00  0.000000E+00       2186.88  0.000000E+00       3110.20       8145.90  0.000000E+00       11014.7       49.9495       10729.8 -1.000000E+10 -1.000000E+10 -1.000000E+10 -1.000000E+10 -1.000000E+10  0.000000E+00  0.000000E+00  0.000000E+00  0.000000E+00  0.000000E+00  0.000000E+00  0.000000E+00  0.000000E+00  0.000000E+00  0.000000E+00  0.000000E+00  0.000000E+00       Funtag_D
      52  4.445339E+05  4.021894E+05       42344.5       11.1096     0.0633609      0.959605      0.200769      0.225824    0.00716865    0.00311377    0.00774653      0.999783       Opttag_D
      52       74.3091       1.76074       1.00000  0.000000E+00       1.00000  0.000000E+00      0.835910       41.5739       1.00000       43.7950  0.000000E+00       3.83507  0.000000E+00       3.83507  0.000000E+00  0.000000E+00      -7.67015       74.5490       2.25418        1       Scatag_D
      52       1684.97       5447.19  0.000000E+00  0.000000E+00       2188.21  0.000000E+00       3109.89       8126.67  0.000000E+00       11012.8       49.9553       10724.8 -1.000000E+10 -1.000000E+10 -1.000000E+10 -1.000000E+10 -1.000000E+10  0.000000E+00  0.000000E+00  0.000000E+00  0.000000E+00  0.000000E+00  0.000000E+00  0.000000E+00  0.000000E+00  0.000000E+00  0.000000E+00  0.000000E+00  0.000000E+00       Funtag_D
      53  4.444901E+05  4.021844E+05       42305.7       10.6053     0.0662807       1.01810      0.200801      0.225848    0.00717913    0.00308852    0.00774088      0.999714       Opttag_D
      53       74.2906       1.75771       1.00000  0.000000E+00       1.00000  0.000000E+00      0.835848       41.5474       1.00000       43.8113  0.000000E+00       3.83644  0.000000E+00       3.83644  0.000000E+00  0.000000E+00      -7.67287       74.5490       2.25418        1       Scatag_D
      53       1682.51       5446.43  0.000000E+00  0.000000E+00       2192.26  0.000000E+00       3109.17       8105.52  0.000000E+00       11008.4       49.9681       10711.4 -1.000000E+10 -1.000000E+10 -1.000000E+10 -1.000000E+10 -1.000000E+10  0.000000E+00  0.000000E+00  0.000000E+00  0.000000E+00  0.000000E+00  0.000000E+00  0.000000E+00  0.000000E+00  0.000000E+00  0.000000E+00  0.000000E+00  0.000000E+00       Funtag_D
      54  4.444666E+05  4.021705E+05       42296.0       13.1518     0.0684060       1.06054      0.200820      0.225879    0.00720800    0.00305197    0.00774394      0.999697       Opttag_D
      54       74.2788       1.75556       1.00000  0.000000E+00       1.00000  0.000000E+00      0.835802       41.5325       1.00000       43.8234  0.000000E+00       3.83729  0.000000E+00       3.83729  0.000000E+00  0.000000E+00      -7.67457       74.5490       2.25418        1       Scatag_D
      54       1683.84       5446.25  0.000000E+00  0.000000E+00       2195.18  0.000000E+00       3109.05       8104.07  0.000000E+00       11005.0       50.0537       10702.6 -1.000000E+10 -1.000000E+10 -1.000000E+10 -1.000000E+10 -1.000000E+10  0.000000E+00  0.000000E+00  0.000000E+00  0.000000E+00  0.000000E+00  0.000000E+00  0.000000E+00  0.000000E+00  0.000000E+00  0.000000E+00  0.000000E+00  0.000000E+00       Funtag_D
      55  4.444310E+05  4.021442E+05       42286.8       12.0841     0.0713941       1.11975      0.200840      0.225924    0.00726302    0.00298849    0.00775308      0.999729       Opttag_D
      55       74.2637       1.75250       1.00000  0.000000E+00       1.00000  0.000000E+00      0.835733       41.5134       1.00000       43.8422  0.000000E+00       3.83846  0.000000E+00       3.83846  0.000000E+00  0.000000E+00      -7.67691       74.5490       2.25418        1       Scatag_D
      55       1687.82       5446.60  0.000000E+00  0.000000E+00       2199.26  0.000000E+00       3109.42       8102.25  0.000000E+00       11000.5       50.1949       10690.8 -1.000000E+10 -1.000000E+10 -1.000000E+10 -1.000000E+10 -1.000000E+10  0.000000E+00  0.000000E+00  0.000000E+00  0.000000E+00  0.000000E+00  0.000000E+00  0.000000E+00  0.000000E+00  0.000000E+00  0.000000E+00  0.000000E+00  0.000000E+00       Funtag_D
      56  4.443945E+05  4.020919E+05       42302.6       18.2890     0.0765230       1.22064      0.200870      0.226008    0.00737198    0.00284731    0.00776984       1.00019       Opttag_D
      56       74.2413       1.74761       1.00000  0.000000E+00       1.00000  0.000000E+00      0.835621       41.4885       1.00000       43.8738  0.000000E+00       3.84065  0.000000E+00       3.84065  0.000000E+00  0.000000E+00      -7.68129       74.5490       2.25418        1       Scatag_D
      56       1695.13       5451.63  0.000000E+00  0.000000E+00       2206.10  0.000000E+00       3112.09       8122.95  0.000000E+00       10994.4       50.4463       10669.9 -1.000000E+10 -1.000000E+10 -1.000000E+10 -1.000000E+10 -1.000000E+10  0.000000E+00  0.000000E+00  0.000000E+00  0.000000E+00  0.000000E+00  0.000000E+00  0.000000E+00  0.000000E+00  0.000000E+00  0.000000E+00  0.000000E+00  0.000000E+00       Funtag_D
      57  4.443530E+05  4.020639E+05       42289.0       11.3416     0.0786006       1.26160      0.200875      0.226052    0.00743030    0.00276445    0.00777785       1.00012       Opttag_D
      57       74.2342       1.74581       1.00000  0.000000E+00       1.00000  0.000000E+00      0.835577       41.4833       1.00000       43.8852  0.000000E+00       3.84127  0.000000E+00       3.84127  0.000000E+00  0.000000E+00      -7.68253       74.5490       2.25418        1       Scatag_D
      57       1698.62       5450.86  0.000000E+00  0.000000E+00       2208.76  0.000000E+00       3114.01       8114.34  0.000000E+00       10992.2       50.5772       10659.7 -1.000000E+10 -1.000000E+10 -1.000000E+10 -1.000000E+10 -1.000000E+10  0.000000E+00  0.000000E+00  0.000000E+00  0.000000E+00  0.000000E+00  0.000000E+00  0.000000E+00  0.000000E+00  0.000000E+00  0.000000E+00  0.000000E+00  0.000000E+00       Funtag_D
      58  4.443280E+05  4.020481E+05       42279.9       10.3845     0.0789313       1.26893      0.200866      0.226061    0.00746336    0.00274477    0.00777621       1.00003       Opttag_D
      58       74.2354       1.74565       1.00000  0.000000E+00       1.00000  0.000000E+00      0.835569       41.4880       1.00000       43.8845  0.000000E+00       3.84135  0.000000E+00       3.84135  0.000000E+00  0.000000E+00      -7.68269       74.5490       2.25418        1       Scatag_D
      58       1697.90       5449.93  0.000000E+00  0.000000E+00       2209.01  0.000000E+00       3115.15       8111.08  0.000000E+00       10991.4       50.5907       10654.8 -1.000000E+10 -1.000000E+10 -1.000000E+10 -1.000000E+10 -1.000000E+10  0.000000E+00  0.000000E+00  0.000000E+00  0.000000E+00  0.000000E+00  0.000000E+00  0.000000E+00  0.000000E+00  0.000000E+00  0.000000E+00  0.000000E+00  0.000000E+00       Funtag_D
      59  4.442845E+05  4.020086E+05       42275.9       13.4774     0.0810384       1.31136      0.200865      0.226102    0.00754575    0.00267346    0.00777731       1.00008       Opttag_D
      59       74.2333       1.74426       1.00000  0.000000E+00       1.00000  0.000000E+00      0.835526       41.4906       1.00000       43.8932  0.000000E+00       3.84183  0.000000E+00       3.84183  0.000000E+00  0.000000E+00      -7.68366       74.5490       2.25418        1       Scatag_D
      59       1698.39       5450.39  0.000000E+00  0.000000E+00       2211.27  0.000000E+00       3118.61       8122.60  0.000000E+00       10989.7       50.6787       10634.2 -1.000000E+10 -1.000000E+10 -1.000000E+10 -1.000000E+10 -1.000000E+10  0.000000E+00  0.000000E+00  0.000000E+00  0.000000E+00  0.000000E+00  0.000000E+00  0.000000E+00  0.000000E+00  0.000000E+00  0.000000E+00  0.000000E+00  0.000000E+00       Funtag_D
      60  4.442500E+05  4.019607E+05       42289.4       22.3622     0.0865749       1.41776      0.200886      0.226225    0.00764562    0.00247569    0.00779481       1.00054       Opttag_D
      60       74.2210       1.74073       1.00000  0.000000E+00       1.00000  0.000000E+00      0.835428       41.4792       1.00000       43.9277  0.000000E+00       3.84338  0.000000E+00       3.84338  0.000000E+00  0.000000E+00      -7.68677       74.5490       2.25418        1       Scatag_D
      60       1706.04       5455.49  0.000000E+00  0.000000E+00       2217.79  0.000000E+00       3125.16       8165.35  0.000000E+00       10990.7       50.9992       10577.9 -1.000000E+10 -1.000000E+10 -1.000000E+10 -1.000000E+10 -1.000000E+10  0.000000E+00  0.000000E+00  0.000000E+00  0.000000E+00  0.000000E+00  0.000000E+00  0.000000E+00  0.000000E+00  0.000000E+00  0.000000E+00  0.000000E+00  0.000000E+00       Funtag_D
      61  4.441988E+05  4.019605E+05       42238.3       13.8301     0.0872030       1.42911      0.200891      0.226236    0.00764592    0.00246904    0.00779199       1.00032       Opttag_D
      61       74.2197       1.74030       1.00000  0.000000E+00       1.00000  0.000000E+00      0.835415       41.4739       1.00000       43.9343  0.000000E+00       3.84346  0.000000E+00       3.84346  0.000000E+00  0.000000E+00      -7.68692       74.5490       2.25418        1       Scatag_D
      61       1704.80       5453.08  0.000000E+00  0.000000E+00       2218.45  0.000000E+00       3124.82       8131.72  0.000000E+00       10990.9       51.0264       10563.5 -1.000000E+10 -1.000000E+10 -1.000000E+10 -1.000000E+10 -1.000000E+10  0.000000E+00  0.000000E+00  0.000000E+00  0.000000E+00  0.000000E+00  0.000000E+00  0.000000E+00  0.000000E+00  0.000000E+00  0.000000E+00  0.000000E+00  0.000000E+00       Funtag_D
      62  4.441489E+05  4.019628E+05       42186.1       11.3508     0.0897411       1.47482      0.200919      0.226294    0.00764113    0.00239092    0.00779654       1.00019       Opttag_D
      62       74.2123       1.73862       1.00000  0.000000E+00       1.00000  0.000000E+00      0.835371       41.4561       1.00000       43.9579  0.000000E+00       3.84407  0.000000E+00       3.84407  0.000000E+00  0.000000E+00      -7.68815       74.5490       2.25418        1       Scatag_D
      62       1706.79       5451.68  0.000000E+00  0.000000E+00       2221.43  0.000000E+00       3124.84       8112.85  0.000000E+00       10993.1       51.1745       10524.2 -1.000000E+10 -1.000000E+10 -1.000000E+10 -1.000000E+10 -1.000000E+10  0.000000E+00  0.000000E+00  0.000000E+00  0.000000E+00  0.000000E+00  0.000000E+00  0.000000E+00  0.000000E+00  0.000000E+00  0.000000E+00  0.000000E+00  0.000000E+00       Funtag_D
      63  4.441153E+05  4.019725E+05       42142.8       10.8393     0.0911022       1.49697      0.200938      0.226336    0.00762095    0.00233025    0.00779509      0.999949       Opttag_D
      63       74.2082       1.73784       1.00000  0.000000E+00       1.00000  0.000000E+00      0.835349       41.4440       1.00000       43.9707  0.000000E+00       3.84447  0.000000E+00       3.84447  0.000000E+00  0.000000E+00      -7.68893       74.5490       2.25418        1       Scatag_D
      63       1706.16       5449.00  0.000000E+00  0.000000E+00       2222.96  0.000000E+00       3123.41       8096.03  0.000000E+00       10994.3       51.2406       10499.7 -1.000000E+10 -1.000000E+10 -1.000000E+10 -1.000000E+10 -1.000000E+10  0.000000E+00  0.000000E+00  0.000000E+00  0.000000E+00  0.000000E+00  0.000000E+00  0.000000E+00  0.000000E+00  0.000000E+00  0.000000E+00  0.000000E+00  0.000000E+00       Funtag_D

 An atom has moved more than     1.50 A from the initial/last
 position - if this was an accepted trial point, probably need to update the
 non-bonded contact list.

      64  4.440977E+05  4.020017E+05       42096.0       21.7398     0.0929519       1.52031      0.200969      0.226412    0.00756003    0.00221725    0.00778825      0.999448       Opttag_D
      64       74.2031       1.73705       1.00000  0.000000E+00       1.00000  0.000000E+00      0.835322       41.4218       1.00000       43.9893  0.000000E+00       3.84527  0.000000E+00       3.84527  0.000000E+00  0.000000E+00      -7.69055       74.5490       2.25418        1       Scatag_D
      64       1703.17       5443.54  0.000000E+00  0.000000E+00       2224.84  0.000000E+00       3117.58       8092.82  0.000000E+00       10995.5       51.2800       10467.3 -1.000000E+10 -1.000000E+10 -1.000000E+10 -1.000000E+10 -1.000000E+10  0.000000E+00  0.000000E+00  0.000000E+00  0.000000E+00  0.000000E+00  0.000000E+00  0.000000E+00  0.000000E+00  0.000000E+00  0.000000E+00  0.000000E+00  0.000000E+00       Funtag_D

 An atom has moved more than     1.50 A from the initial/last
 position - need to update the non-bonded contact list.


 ############################################################################################################
 ###################################################### setting up contact terms
 ############################################################################################################

      65  4.440524E+05  4.020032E+05       42049.2       9.18441     0.0926358       1.51226      0.200966      0.226408    0.00755694    0.00222640    0.00778250      0.999205       Opttag_D
      65       74.2048       1.73744       1.00000  0.000000E+00       1.00000  0.000000E+00      0.835327       41.4253       1.00000       43.9839  0.000000E+00       3.84508  0.000000E+00       3.84508  0.000000E+00  0.000000E+00      -7.69016       74.5490       2.25418        1       Scatag_D
      65       1700.65       5440.90  0.000000E+00  0.000000E+00       2224.34  0.000000E+00       3116.21       8055.36  0.000000E+00       10994.5       51.2399       10465.9 -1.000000E+10 -1.000000E+10 -1.000000E+10 -1.000000E+10 -1.000000E+10  0.000000E+00  0.000000E+00  0.000000E+00  0.000000E+00  0.000000E+00  0.000000E+00  0.000000E+00  0.000000E+00  0.000000E+00  0.000000E+00  0.000000E+00  0.000000E+00       Funtag_D
      66  4.440386E+05  4.020031E+05       42035.5       7.78867     0.0920004       1.49842      0.200957      0.226394    0.00755714    0.00224542    0.00777698      0.999116       Opttag_D
      66       74.2077       1.73800       1.00000  0.000000E+00       1.00000  0.000000E+00      0.835338       41.4308       1.00000       43.9767  0.000000E+00       3.84483  0.000000E+00       3.84483  0.000000E+00  0.000000E+00      -7.68965       74.5490       2.25418        1       Scatag_D
      66       1698.24       5439.92  0.000000E+00  0.000000E+00       2223.47  0.000000E+00       3114.71       8048.15  0.000000E+00       10993.2       51.1696       10466.6 -1.000000E+10 -1.000000E+10 -1.000000E+10 -1.000000E+10 -1.000000E+10  0.000000E+00  0.000000E+00  0.000000E+00  0.000000E+00  0.000000E+00  0.000000E+00  0.000000E+00  0.000000E+00  0.000000E+00  0.000000E+00  0.000000E+00  0.000000E+00       Funtag_D
      67  4.440062E+05  4.020116E+05       41994.6       8.81515     0.0916377       1.47942      0.200949      0.226390    0.00753946    0.00226104    0.00776592      0.998799       Opttag_D
      67       74.2135       1.73907       1.00000  0.000000E+00       1.00000  0.000000E+00      0.835349       41.4372       1.00000       43.9682  0.000000E+00       3.84443  0.000000E+00       3.84443  0.000000E+00  0.000000E+00      -7.68885       74.5490       2.25418        1       Scatag_D
      67       1693.41       5436.47  0.000000E+00  0.000000E+00       2222.50  0.000000E+00       3109.51       8031.32  0.000000E+00       10991.0       51.0115       10459.4 -1.000000E+10 -1.000000E+10 -1.000000E+10 -1.000000E+10 -1.000000E+10  0.000000E+00  0.000000E+00  0.000000E+00  0.000000E+00  0.000000E+00  0.000000E+00  0.000000E+00  0.000000E+00  0.000000E+00  0.000000E+00  0.000000E+00  0.000000E+00       Funtag_D
      68  4.439855E+05  4.020180E+05       41967.5       12.4848     0.0917671       1.46762      0.200945      0.226401    0.00752608    0.00223992    0.00776164      0.998519       Opttag_D
      68       74.2194       1.73998       1.00000  0.000000E+00       1.00000  0.000000E+00      0.835352       41.4430       1.00000       43.9642  0.000000E+00       3.84400  0.000000E+00       3.84400  0.000000E+00  0.000000E+00      -7.68800       74.5490       2.25418        2       Scatag_D
      68       1691.55       5433.43  0.000000E+00  0.000000E+00       2222.06  0.000000E+00       3105.25       8023.02  0.000000E+00       10989.2       50.9023       10452.1 -1.000000E+10 -1.000000E+10 -1.000000E+10 -1.000000E+10 -1.000000E+10  0.000000E+00  0.000000E+00  0.000000E+00  0.000000E+00  0.000000E+00  0.000000E+00  0.000000E+00  0.000000E+00  0.000000E+00  0.000000E+00  0.000000E+00  0.000000E+00       Funtag_D
      69  4.439607E+05  4.020253E+05       41935.5       8.87968     0.0922134       1.46174      0.200944      0.226411    0.00751090    0.00223908    0.00775971      0.998259       Opttag_D
      69       74.2246       1.74073       1.00000  0.000000E+00       1.00000  0.000000E+00      0.835353       41.4468       1.00000       43.9626  0.000000E+00       3.84372  0.000000E+00       3.84372  0.000000E+00  0.000000E+00      -7.68745       74.5490       2.25418        1       Scatag_D
      69       1690.71       5430.59  0.000000E+00  0.000000E+00       2222.05  0.000000E+00       3101.77       8004.48  0.000000E+00       10988.2       50.8232       10446.9 -1.000000E+10 -1.000000E+10 -1.000000E+10 -1.000000E+10 -1.000000E+10  0.000000E+00  0.000000E+00  0.000000E+00  0.000000E+00  0.000000E+00  0.000000E+00  0.000000E+00  0.000000E+00  0.000000E+00  0.000000E+00  0.000000E+00  0.000000E+00       Funtag_D
      70  4.439372E+05  4.020276E+05       41909.5       8.40219     0.0929611       1.45848      0.200941      0.226423    0.00750590    0.00223322    0.00776069      0.997942       Opttag_D
      70       74.2312       1.74145       1.00000  0.000000E+00       1.00000  0.000000E+00      0.835352       41.4513       1.00000       43.9626  0.000000E+00       3.84340  0.000000E+00       3.84340  0.000000E+00  0.000000E+00      -7.68680       74.5490       2.25418        1       Scatag_D
      70       1691.14       5427.15  0.000000E+00  0.000000E+00       2222.32  0.000000E+00       3098.80       7991.32  0.000000E+00       10986.7       50.7980       10441.3 -1.000000E+10 -1.000000E+10 -1.000000E+10 -1.000000E+10 -1.000000E+10  0.000000E+00  0.000000E+00  0.000000E+00  0.000000E+00  0.000000E+00  0.000000E+00  0.000000E+00  0.000000E+00  0.000000E+00  0.000000E+00  0.000000E+00  0.000000E+00       Funtag_D
      71  4.439100E+05  4.020270E+05       41883.0       11.1688     0.0940976       1.45199      0.200937      0.226440    0.00750720    0.00221731    0.00776712      0.997522       Opttag_D
      71       74.2432       1.74279       1.00000  0.000000E+00       1.00000  0.000000E+00      0.835351       41.4620       1.00000       43.9610  0.000000E+00       3.84265  0.000000E+00       3.84265  0.000000E+00  0.000000E+00      -7.68529       74.5490       2.25418        1       Scatag_D
      71       1693.94       5422.59  0.000000E+00  0.000000E+00       2222.75  0.000000E+00       3095.41       7981.49  0.000000E+00       10984.2       50.8133       10431.8 -1.000000E+10 -1.000000E+10 -1.000000E+10 -1.000000E+10 -1.000000E+10  0.000000E+00  0.000000E+00  0.000000E+00  0.000000E+00  0.000000E+00  0.000000E+00  0.000000E+00  0.000000E+00  0.000000E+00  0.000000E+00  0.000000E+00  0.000000E+00       Funtag_D
      72  4.438787E+05  4.020206E+05       41858.1       10.7504     0.0951918       1.44288      0.200926      0.226451    0.00752058    0.00221521    0.00777935      0.997197       Opttag_D
      72       74.2573       1.74417       1.00000  0.000000E+00       1.00000  0.000000E+00      0.835353       41.4740       1.00000       43.9588  0.000000E+00       3.84182  0.000000E+00       3.84182  0.000000E+00  0.000000E+00      -7.68364       74.5490       2.25418        1       Scatag_D
      72       1699.28       5419.05  0.000000E+00  0.000000E+00       2223.12  0.000000E+00       3093.24       7975.21  0.000000E+00       10981.6       50.8678       10415.8 -1.000000E+10 -1.000000E+10 -1.000000E+10 -1.000000E+10 -1.000000E+10  0.000000E+00  0.000000E+00  0.000000E+00  0.000000E+00  0.000000E+00  0.000000E+00  0.000000E+00  0.000000E+00  0.000000E+00  0.000000E+00  0.000000E+00  0.000000E+00       Funtag_D
      73  4.438393E+05  4.020083E+05       41831.0       10.8336     0.0961269       1.43144      0.200911      0.226450    0.00754635    0.00220893    0.00779313      0.996985       Opttag_D
      73       74.2724       1.74558       1.00000  0.000000E+00       1.00000  0.000000E+00      0.835357       41.4876       1.00000       43.9555  0.000000E+00       3.84092  0.000000E+00       3.84092  0.000000E+00  0.000000E+00      -7.68184       74.5490       2.25418        1       Scatag_D
      73       1705.30       5416.74  0.000000E+00  0.000000E+00       2223.39  0.000000E+00       3092.49       7975.20  0.000000E+00       10978.7       50.9423       10388.2 -1.000000E+10 -1.000000E+10 -1.000000E+10 -1.000000E+10 -1.000000E+10  0.000000E+00  0.000000E+00  0.000000E+00  0.000000E+00  0.000000E+00  0.000000E+00  0.000000E+00  0.000000E+00  0.000000E+00  0.000000E+00  0.000000E+00  0.000000E+00       Funtag_D
      74  4.438028E+05  4.019892E+05       41813.5       18.4024     0.0983357       1.40038      0.200880      0.226431    0.00758610    0.00225051    0.00783280      0.996994       Opttag_D
      74       74.3101       1.74938       1.00000  0.000000E+00       1.00000  0.000000E+00      0.835375       41.5224       1.00000       43.9464  0.000000E+00       3.83860  0.000000E+00       3.83860  0.000000E+00  0.000000E+00      -7.67719       74.5490       2.25418        1       Scatag_D
      74       1722.71       5416.84  0.000000E+00  0.000000E+00       2224.08  0.000000E+00       3094.26       8001.28  0.000000E+00       10973.4       51.0720       10329.9 -1.000000E+10 -1.000000E+10 -1.000000E+10 -1.000000E+10 -1.000000E+10  0.000000E+00  0.000000E+00  0.000000E+00  0.000000E+00  0.000000E+00  0.000000E+00  0.000000E+00  0.000000E+00  0.000000E+00  0.000000E+00  0.000000E+00  0.000000E+00       Funtag_D
      75  4.437429E+05  4.019794E+05       41763.5       13.9254     0.0973727       1.40076      0.200880      0.226405    0.00760657    0.00226421    0.00779848      0.996618       Opttag_D
      75       74.3037       1.74838       1.00000  0.000000E+00       1.00000  0.000000E+00      0.835369       41.5149       1.00000       43.9462  0.000000E+00       3.83922  0.000000E+00       3.83922  0.000000E+00  0.000000E+00      -7.67843       74.5490       2.25418        1       Scatag_D
      75       1707.64       5412.75  0.000000E+00  0.000000E+00       2223.26  0.000000E+00       3094.43       7981.79  0.000000E+00       10971.5       51.0066       10321.2 -1.000000E+10 -1.000000E+10 -1.000000E+10 -1.000000E+10 -1.000000E+10  0.000000E+00  0.000000E+00  0.000000E+00  0.000000E+00  0.000000E+00  0.000000E+00  0.000000E+00  0.000000E+00  0.000000E+00  0.000000E+00  0.000000E+00  0.000000E+00       Funtag_D
      76  4.437163E+05  4.019801E+05       41736.2       7.98206     0.0968876       1.40132      0.200884      0.226385    0.00760497    0.00231332    0.00778320      0.996448       Opttag_D
      76       74.2996       1.74789       1.00000  0.000000E+00       1.00000  0.000000E+00      0.835366       41.5104       1.00000       43.9460  0.000000E+00       3.83953  0.000000E+00       3.83953  0.000000E+00  0.000000E+00      -7.67905       74.5490       2.25418        1       Scatag_D
      76       1700.96       5410.91  0.000000E+00  0.000000E+00       2222.84  0.000000E+00       3094.45       7965.51  0.000000E+00       10970.7       50.9385       10319.9 -1.000000E+10 -1.000000E+10 -1.000000E+10 -1.000000E+10 -1.000000E+10  0.000000E+00  0.000000E+00  0.000000E+00  0.000000E+00  0.000000E+00  0.000000E+00  0.000000E+00  0.000000E+00  0.000000E+00  0.000000E+00  0.000000E+00  0.000000E+00       Funtag_D
      77  4.436931E+05  4.019820E+05       41711.1       7.37930     0.0971545       1.39259      0.200882      0.226366    0.00760128    0.00235682    0.00777775      0.996347       Opttag_D
      77       74.3066       1.74860       1.00000  0.000000E+00       1.00000  0.000000E+00      0.835371       41.5140       1.00000       43.9445  0.000000E+00       3.83932  0.000000E+00       3.83932  0.000000E+00  0.000000E+00      -7.67864       74.5490       2.25418        1       Scatag_D
      77       1698.58       5409.82  0.000000E+00  0.000000E+00       2222.66  0.000000E+00       3094.82       7958.91  0.000000E+00       10968.2       50.8708       10307.2 -1.000000E+10 -1.000000E+10 -1.000000E+10 -1.000000E+10 -1.000000E+10  0.000000E+00  0.000000E+00  0.000000E+00  0.000000E+00  0.000000E+00  0.000000E+00  0.000000E+00  0.000000E+00  0.000000E+00  0.000000E+00  0.000000E+00  0.000000E+00       Funtag_D
      78  4.436723E+05  4.019900E+05       41682.3       9.96113     0.0977159       1.37830      0.200883      0.226343    0.00758450    0.00241837    0.00777206      0.996243       Opttag_D
      78       74.3183       1.74984       1.00000  0.000000E+00       1.00000  0.000000E+00      0.835382       41.5183       1.00000       43.9435  0.000000E+00       3.83903  0.000000E+00       3.83903  0.000000E+00  0.000000E+00      -7.67806       74.5490       2.25418        1       Scatag_D
      78       1696.10       5408.69  0.000000E+00  0.000000E+00       2222.40  0.000000E+00       3095.08       7950.69  0.000000E+00       10964.1       50.7397       10294.5 -1.000000E+10 -1.000000E+10 -1.000000E+10 -1.000000E+10 -1.000000E+10  0.000000E+00  0.000000E+00  0.000000E+00  0.000000E+00  0.000000E+00  0.000000E+00  0.000000E+00  0.000000E+00  0.000000E+00  0.000000E+00  0.000000E+00  0.000000E+00       Funtag_D
      79  4.436532E+05  4.019973E+05       41655.9       8.20983     0.0980475       1.37111      0.200883      0.226336    0.00756922    0.00243602    0.00776632      0.996108       Opttag_D
      79       74.3237       1.75045       1.00000  0.000000E+00       1.00000  0.000000E+00      0.835390       41.5183       1.00000       43.9435  0.000000E+00       3.83900  0.000000E+00       3.83900  0.000000E+00  0.000000E+00      -7.67801       74.5490       2.25418        1       Scatag_D
      79       1693.59       5407.22  0.000000E+00  0.000000E+00       2222.24  0.000000E+00       3094.45       7938.47  0.000000E+00       10961.5       50.6460       10287.8 -1.000000E+10 -1.000000E+10 -1.000000E+10 -1.000000E+10 -1.000000E+10  0.000000E+00  0.000000E+00  0.000000E+00  0.000000E+00  0.000000E+00  0.000000E+00  0.000000E+00  0.000000E+00  0.000000E+00  0.000000E+00  0.000000E+00  0.000000E+00       Funtag_D
      80  4.436391E+05  4.020005E+05       41638.6       6.52619     0.0981150       1.36928      0.200884      0.226337    0.00756263    0.00244075    0.00776449      0.996015       Opttag_D
      80       74.3245       1.75050       1.00000  0.000000E+00       1.00000  0.000000E+00      0.835394       41.5160       1.00000       43.9438  0.000000E+00       3.83914  0.000000E+00       3.83914  0.000000E+00  0.000000E+00      -7.67828       74.5490       2.25418        1       Scatag_D
      80       1692.79       5406.21  0.000000E+00  0.000000E+00       2222.08  0.000000E+00       3093.73       7931.14  0.000000E+00       10960.2       50.6098       10281.9 -1.000000E+10 -1.000000E+10 -1.000000E+10 -1.000000E+10 -1.000000E+10  0.000000E+00  0.000000E+00  0.000000E+00  0.000000E+00  0.000000E+00  0.000000E+00  0.000000E+00  0.000000E+00  0.000000E+00  0.000000E+00  0.000000E+00  0.000000E+00       Funtag_D
      81  4.436250E+05  4.020012E+05       41623.8       7.30753     0.0982369       1.36485      0.200880      0.226337    0.00756113    0.00244553    0.00776556      0.995881       Opttag_D
      81       74.3270       1.75071       1.00000  0.000000E+00       1.00000  0.000000E+00      0.835400       41.5150       1.00000       43.9431  0.000000E+00       3.83913  0.000000E+00       3.83913  0.000000E+00  0.000000E+00      -7.67826       74.5490       2.25418        1       Scatag_D
      81       1693.26       5404.75  0.000000E+00  0.000000E+00       2221.67  0.000000E+00       3092.82       7928.46  0.000000E+00       10957.6       50.6022       10274.6 -1.000000E+10 -1.000000E+10 -1.000000E+10 -1.000000E+10 -1.000000E+10  0.000000E+00  0.000000E+00  0.000000E+00  0.000000E+00  0.000000E+00  0.000000E+00  0.000000E+00  0.000000E+00  0.000000E+00  0.000000E+00  0.000000E+00  0.000000E+00       Funtag_D
      82  4.436146E+05  4.019988E+05       41615.8       7.46287     0.0984148       1.35853      0.200875      0.226329    0.00756603    0.00246994    0.00776632      0.995764       Opttag_D
      82       74.3317       1.75112       1.00000  0.000000E+00       1.00000  0.000000E+00      0.835406       41.5171       1.00000       43.9406  0.000000E+00       3.83902  0.000000E+00       3.83902  0.000000E+00  0.000000E+00      -7.67803       74.5490       2.25418        1       Scatag_D
      82       1693.59       5403.49  0.000000E+00  0.000000E+00       2221.24  0.000000E+00       3092.62       7927.29  0.000000E+00       10955.1       50.6069       10271.8 -1.000000E+10 -1.000000E+10 -1.000000E+10 -1.000000E+10 -1.000000E+10  0.000000E+00  0.000000E+00  0.000000E+00  0.000000E+00  0.000000E+00  0.000000E+00  0.000000E+00  0.000000E+00  0.000000E+00  0.000000E+00  0.000000E+00  0.000000E+00       Funtag_D
      83  4.436053E+05  4.019943E+05       41610.9       6.09848     0.0985044       1.35490      0.200870      0.226327    0.00757541    0.00248005    0.00776513      0.995696       Opttag_D
      83       74.3348       1.75138       1.00000  0.000000E+00       1.00000  0.000000E+00      0.835409       41.5199       1.00000       43.9387  0.000000E+00       3.83886  0.000000E+00       3.83886  0.000000E+00  0.000000E+00      -7.67771       74.5490       2.25418        1       Scatag_D
      83       1693.07       5402.75  0.000000E+00  0.000000E+00       2220.99  0.000000E+00       3092.71       7926.25  0.000000E+00       10953.9       50.6247       10270.6 -1.000000E+10 -1.000000E+10 -1.000000E+10 -1.000000E+10 -1.000000E+10  0.000000E+00  0.000000E+00  0.000000E+00  0.000000E+00  0.000000E+00  0.000000E+00  0.000000E+00  0.000000E+00  0.000000E+00  0.000000E+00  0.000000E+00  0.000000E+00       Funtag_D
      84  4.435889E+05  4.019819E+05       41607.0       6.54738     0.0988556       1.34172      0.200852      0.226326    0.00760128    0.00249525    0.00776163      0.995577       Opttag_D
      84       74.3460       1.75243       1.00000  0.000000E+00       1.00000  0.000000E+00      0.835417       41.5305       1.00000       43.9313  0.000000E+00       3.83826  0.000000E+00       3.83826  0.000000E+00  0.000000E+00      -7.67653       74.5490       2.25418        1       Scatag_D
      84       1691.55       5401.45  0.000000E+00  0.000000E+00       2220.16  0.000000E+00       3093.10       7931.42  0.000000E+00       10949.6       50.6400       10269.0 -1.000000E+10 -1.000000E+10 -1.000000E+10 -1.000000E+10 -1.000000E+10  0.000000E+00  0.000000E+00  0.000000E+00  0.000000E+00  0.000000E+00  0.000000E+00  0.000000E+00  0.000000E+00  0.000000E+00  0.000000E+00  0.000000E+00  0.000000E+00       Funtag_D
      85  4.435804E+05  4.019784E+05       41602.0       9.79937     0.0991475       1.33110      0.200843      0.226328    0.00760857    0.00252857    0.00775811      0.995525       Opttag_D
      85       74.3543       1.75324       1.00000  0.000000E+00       1.00000  0.000000E+00      0.835425       41.5358       1.00000       43.9251  0.000000E+00       3.83804  0.000000E+00       3.83804  0.000000E+00  0.000000E+00      -7.67608       74.5490       2.25418        1       Scatag_D
      85       1690.01       5400.89  0.000000E+00  0.000000E+00       2219.46  0.000000E+00       3093.03       7934.75  0.000000E+00       10945.6       50.5510       10267.7 -1.000000E+10 -1.000000E+10 -1.000000E+10 -1.000000E+10 -1.000000E+10  0.000000E+00  0.000000E+00  0.000000E+00  0.000000E+00  0.000000E+00  0.000000E+00  0.000000E+00  0.000000E+00  0.000000E+00  0.000000E+00  0.000000E+00  0.000000E+00       Funtag_D
      86  4.435697E+05  4.019772E+05       41592.5       7.06722     0.0992513       1.32726      0.200840      0.226332    0.00761117    0.00252283    0.00775586      0.995489       Opttag_D
      86       74.3573       1.75353       1.00000  0.000000E+00       1.00000  0.000000E+00      0.835427       41.5377       1.00000       43.9227  0.000000E+00       3.83798  0.000000E+00       3.83798  0.000000E+00  0.000000E+00      -7.67596       74.5490       2.25418        1       Scatag_D
      86       1689.03       5400.50  0.000000E+00  0.000000E+00       2219.21  0.000000E+00       3092.95       7929.61  0.000000E+00       10944.1       50.4974       10266.7 -1.000000E+10 -1.000000E+10 -1.000000E+10 -1.000000E+10 -1.000000E+10  0.000000E+00  0.000000E+00  0.000000E+00  0.000000E+00  0.000000E+00  0.000000E+00  0.000000E+00  0.000000E+00  0.000000E+00  0.000000E+00  0.000000E+00  0.000000E+00       Funtag_D
      87  4.435495E+05  4.019752E+05       41574.3       5.86020     0.0996546       1.31292      0.200828      0.226349    0.00761526    0.00251942    0.00775240      0.995416       Opttag_D
      87       74.3679       1.75465       1.00000  0.000000E+00       1.00000  0.000000E+00      0.835435       41.5442       1.00000       43.9133  0.000000E+00       3.83778  0.000000E+00       3.83778  0.000000E+00  0.000000E+00      -7.67555       74.5490       2.25418        1       Scatag_D
      87       1687.52       5399.71  0.000000E+00  0.000000E+00       2218.29  0.000000E+00       3092.59       7923.57  0.000000E+00       10938.4       50.3010       10263.9 -1.000000E+10 -1.000000E+10 -1.000000E+10 -1.000000E+10 -1.000000E+10  0.000000E+00  0.000000E+00  0.000000E+00  0.000000E+00  0.000000E+00  0.000000E+00  0.000000E+00  0.000000E+00  0.000000E+00  0.000000E+00  0.000000E+00  0.000000E+00       Funtag_D
      88  4.435391E+05  4.019736E+05       41565.5       6.40313     0.0999148       1.30419      0.200818      0.226358    0.00761866    0.00251852    0.00775303      0.995317       Opttag_D
      88       74.3739       1.75526       1.00000  0.000000E+00       1.00000  0.000000E+00      0.835441       41.5475       1.00000       43.9076  0.000000E+00       3.83773  0.000000E+00       3.83773  0.000000E+00  0.000000E+00      -7.67545       74.5490       2.25418        1       Scatag_D
      88       1687.80       5398.64  0.000000E+00  0.000000E+00       2217.73  0.000000E+00       3092.36       7921.19  0.000000E+00       10935.2       50.2519       10262.3 -1.000000E+10 -1.000000E+10 -1.000000E+10 -1.000000E+10 -1.000000E+10  0.000000E+00  0.000000E+00  0.000000E+00  0.000000E+00  0.000000E+00  0.000000E+00  0.000000E+00  0.000000E+00  0.000000E+00  0.000000E+00  0.000000E+00  0.000000E+00       Funtag_D
      89  4.435213E+05  4.019719E+05       41549.4       8.29526      0.100722       1.34113      0.200804      0.226386    0.00762225    0.00253539    0.00775863      0.995093       Opttag_D
      89       74.3900       1.75695       1.00000  0.000000E+00       1.00000  0.000000E+00      0.835456       41.5556       1.00000       43.8914  0.000000E+00       3.83767  0.000000E+00       3.83767  0.000000E+00  0.000000E+00      -7.67534       74.5490       2.25418        1       Scatag_D
      89       1690.24       5396.20  0.000000E+00  0.000000E+00       2216.22  0.000000E+00       3091.84       7917.24  0.000000E+00       10926.4       50.1999       10261.1 -1.000000E+10 -1.000000E+10 -1.000000E+10 -1.000000E+10 -1.000000E+10  0.000000E+00  0.000000E+00  0.000000E+00  0.000000E+00  0.000000E+00  0.000000E+00  0.000000E+00  0.000000E+00  0.000000E+00  0.000000E+00  0.000000E+00  0.000000E+00       Funtag_D
      90  4.435081E+05  4.019730E+05       41535.1       7.98215      0.100918       1.34890      0.200799      0.226393    0.00761995    0.00252869    0.00775701      0.994992       Opttag_D
      90       74.3918       1.75721       1.00000  0.000000E+00       1.00000  0.000000E+00      0.835459       41.5559       1.00000       43.8875  0.000000E+00       3.83797  0.000000E+00       3.83797  0.000000E+00  0.000000E+00      -7.67594       74.5490       2.25418        1       Scatag_D
      90       1689.53       5395.11  0.000000E+00  0.000000E+00       2215.83  0.000000E+00       3091.39       7911.11  0.000000E+00       10924.0       50.3035       10257.8 -1.000000E+10 -1.000000E+10 -1.000000E+10 -1.000000E+10 -1.000000E+10  0.000000E+00  0.000000E+00  0.000000E+00  0.000000E+00  0.000000E+00  0.000000E+00  0.000000E+00  0.000000E+00  0.000000E+00  0.000000E+00  0.000000E+00  0.000000E+00       Funtag_D
      91  4.434978E+05  4.019739E+05       41523.9       6.49868      0.100916       1.34777      0.200801      0.226395    0.00761816    0.00252396    0.00775365      0.994946       Opttag_D
      91       74.3901       1.75704       1.00000  0.000000E+00       1.00000  0.000000E+00      0.835458       41.5540       1.00000       43.8876  0.000000E+00       3.83822  0.000000E+00       3.83822  0.000000E+00  0.000000E+00      -7.67643       74.5490       2.25418        1       Scatag_D
      91       1688.07       5394.61  0.000000E+00  0.000000E+00       2215.78  0.000000E+00       3091.03       7905.83  0.000000E+00       10923.7       50.3911       10254.5 -1.000000E+10 -1.000000E+10 -1.000000E+10 -1.000000E+10 -1.000000E+10  0.000000E+00  0.000000E+00  0.000000E+00  0.000000E+00  0.000000E+00  0.000000E+00  0.000000E+00  0.000000E+00  0.000000E+00  0.000000E+00  0.000000E+00  0.000000E+00       Funtag_D
      92  4.434803E+05  4.019770E+05       41503.3       6.32334      0.101116       1.35574      0.200803      0.226403    0.00761147    0.00252121    0.00774781      0.994928       Opttag_D
      92       74.3891       1.75700       1.00000  0.000000E+00       1.00000  0.000000E+00      0.835458       41.5509       1.00000       43.8850  0.000000E+00       3.83878  0.000000E+00       3.83878  0.000000E+00  0.000000E+00      -7.67755       74.5490       2.25418        1       Scatag_D
      92       1685.53       5394.41  0.000000E+00  0.000000E+00       2215.34  0.000000E+00       3089.98       7897.67  0.000000E+00       10921.2       50.5554       10248.6 -1.000000E+10 -1.000000E+10 -1.000000E+10 -1.000000E+10 -1.000000E+10  0.000000E+00  0.000000E+00  0.000000E+00  0.000000E+00  0.000000E+00  0.000000E+00  0.000000E+00  0.000000E+00  0.000000E+00  0.000000E+00  0.000000E+00  0.000000E+00       Funtag_D
      93  4.434672E+05  4.019829E+05       41484.2       9.92909      0.101646       1.38027      0.200801      0.226426    0.00759918    0.00248657    0.00774273      0.995102       Opttag_D
      93       74.3924       1.75755       1.00000  0.000000E+00       1.00000  0.000000E+00      0.835465       41.5506       1.00000       43.8778  0.000000E+00       3.83925  0.000000E+00       3.83925  0.000000E+00  0.000000E+00      -7.67851       74.5490       2.25418        1       Scatag_D
      93       1683.32       5396.30  0.000000E+00  0.000000E+00       2214.42  0.000000E+00       3088.63       7890.25  0.000000E+00       10916.0       50.6111       10244.7 -1.000000E+10 -1.000000E+10 -1.000000E+10 -1.000000E+10 -1.000000E+10  0.000000E+00  0.000000E+00  0.000000E+00  0.000000E+00  0.000000E+00  0.000000E+00  0.000000E+00  0.000000E+00  0.000000E+00  0.000000E+00  0.000000E+00  0.000000E+00       Funtag_D
      94  4.434503E+05  4.019862E+05       41464.1       6.91382      0.102138       1.40337      0.200796      0.226438    0.00759249    0.00249501    0.00774126      0.995054       Opttag_D
      94       74.3962       1.75804       1.00000  0.000000E+00       1.00000  0.000000E+00      0.835473       41.5502       1.00000       43.8720  0.000000E+00       3.83949  0.000000E+00       3.83949  0.000000E+00  0.000000E+00      -7.67898       74.5490       2.25418        1       Scatag_D
      94       1682.68       5395.78  0.000000E+00  0.000000E+00       2213.69  0.000000E+00       3087.65       7879.04  0.000000E+00       10911.7       50.5721       10243.0 -1.000000E+10 -1.000000E+10 -1.000000E+10 -1.000000E+10 -1.000000E+10  0.000000E+00  0.000000E+00  0.000000E+00  0.000000E+00  0.000000E+00  0.000000E+00  0.000000E+00  0.000000E+00  0.000000E+00  0.000000E+00  0.000000E+00  0.000000E+00       Funtag_D
      95  4.434304E+05  4.019903E+05       41440.1       6.08209      0.103036       1.44590      0.200789      0.226455    0.00758380    0.00250500    0.00774237      0.995060       Opttag_D
      95       74.4041       1.75904       1.00000  0.000000E+00       1.00000  0.000000E+00      0.835487       41.5524       1.00000       43.8616  0.000000E+00       3.83961  0.000000E+00       3.83961  0.000000E+00  0.000000E+00      -7.67922       74.5490       2.25418        1       Scatag_D
      95       1683.16       5395.85  0.000000E+00  0.000000E+00       2212.54  0.000000E+00       3086.50       7866.85  0.000000E+00       10904.8       50.4138       10240.0 -1.000000E+10 -1.000000E+10 -1.000000E+10 -1.000000E+10 -1.000000E+10  0.000000E+00  0.000000E+00  0.000000E+00  0.000000E+00  0.000000E+00  0.000000E+00  0.000000E+00  0.000000E+00  0.000000E+00  0.000000E+00  0.000000E+00  0.000000E+00       Funtag_D
      96  4.434176E+05  4.019892E+05       41428.4       9.50928      0.103931       1.48745      0.200779      0.226463    0.00758610    0.00250165    0.00774608      0.995129       Opttag_D
      96       74.4110       1.75986       1.00000  0.000000E+00       1.00000  0.000000E+00      0.835503       41.5554       1.00000       43.8526  0.000000E+00       3.83961  0.000000E+00       3.83961  0.000000E+00  0.000000E+00      -7.67921       74.5490       2.25418        1       Scatag_D
      96       1684.77       5396.59  0.000000E+00  0.000000E+00       2211.59  0.000000E+00       3086.46       7863.96  0.000000E+00       10898.6       50.3548       10236.1 -1.000000E+10 -1.000000E+10 -1.000000E+10 -1.000000E+10 -1.000000E+10  0.000000E+00  0.000000E+00  0.000000E+00  0.000000E+00  0.000000E+00  0.000000E+00  0.000000E+00  0.000000E+00  0.000000E+00  0.000000E+00  0.000000E+00  0.000000E+00       Funtag_D
      97  4.434016E+05  4.019862E+05       41415.4       6.94991      0.104503       1.51460      0.200773      0.226461    0.00759229    0.00250978    0.00774828      0.994999       Opttag_D
      97       74.4153       1.76039       1.00000  0.000000E+00       1.00000  0.000000E+00      0.835513       41.5596       1.00000       43.8465  0.000000E+00       3.83950  0.000000E+00       3.83950  0.000000E+00  0.000000E+00      -7.67901       74.5490       2.25418        1       Scatag_D
      97       1685.73       5395.18  0.000000E+00  0.000000E+00       2211.07  0.000000E+00       3087.01       7859.16  0.000000E+00       10895.0       50.3338       10231.9 -1.000000E+10 -1.000000E+10 -1.000000E+10 -1.000000E+10 -1.000000E+10  0.000000E+00  0.000000E+00  0.000000E+00  0.000000E+00  0.000000E+00  0.000000E+00  0.000000E+00  0.000000E+00  0.000000E+00  0.000000E+00  0.000000E+00  0.000000E+00       Funtag_D
      98  4.433797E+05  4.019743E+05       41405.5       6.48692      0.105366       1.55670      0.200759      0.226451    0.00761726    0.00251995    0.00775214      0.994890       Opttag_D
      98       74.4208       1.76093       1.00000  0.000000E+00       1.00000  0.000000E+00      0.835528       41.5669       1.00000       43.8382  0.000000E+00       3.83931  0.000000E+00       3.83931  0.000000E+00  0.000000E+00      -7.67861       74.5490       2.25418        1       Scatag_D
      98       1687.41       5394.00  0.000000E+00  0.000000E+00       2210.43  0.000000E+00       3089.00       7860.45  0.000000E+00       10889.9       50.4195       10223.8 -1.000000E+10 -1.000000E+10 -1.000000E+10 -1.000000E+10 -1.000000E+10  0.000000E+00  0.000000E+00  0.000000E+00  0.000000E+00  0.000000E+00  0.000000E+00  0.000000E+00  0.000000E+00  0.000000E+00  0.000000E+00  0.000000E+00  0.000000E+00       Funtag_D
      99  4.433647E+05  4.019630E+05       41401.7       9.90217      0.106232       1.59927      0.200749      0.226429    0.00764073    0.00254502    0.00775759      0.994833       Opttag_D
      99       74.4250       1.76141       1.00000  0.000000E+00       1.00000  0.000000E+00      0.835544       41.5747       1.00000       43.8304  0.000000E+00       3.83925  0.000000E+00       3.83925  0.000000E+00  0.000000E+00      -7.67850       74.5490       2.25418        1       Scatag_D
      99       1689.79       5393.39  0.000000E+00  0.000000E+00       2209.95  0.000000E+00       3091.25       7864.63  0.000000E+00       10885.1       50.5718       10217.1 -1.000000E+10 -1.000000E+10 -1.000000E+10 -1.000000E+10 -1.000000E+10  0.000000E+00  0.000000E+00  0.000000E+00  0.000000E+00  0.000000E+00  0.000000E+00  0.000000E+00  0.000000E+00  0.000000E+00  0.000000E+00  0.000000E+00  0.000000E+00       Funtag_D

   MaxLik Scaling Cycle 0106

   Wilson plot

   Statistics in resolution bins after refinement cycle 0003
```


|  |  |
| --- | --- |
|  | - Rxpct factors plot- Rxpct factors table- <Log-Lik Gain>- Ln Sigma A- Correlation Coefficients |


```
   Iters     Tot_Funct    Xray_Funct    Geom_Funct          Grms     rmsD2init     maxD2init             R         Rfree           LLG       LLGfree     rms(bond)    rms(angle)
   Iters             K   B_IMPF_FRAG        K_MISS   B_TEMP_MISS   K_IMPF_MISS   B_IMPF_MISS        K_SOLV   B_TEMP_SOLV   K_IMPF_SOLV   B_IMPF_SOLV        B_TEMP     B_TEMP_11     B_TEMP_12     B_TEMP_22     B_TEMP_13     B_TEMP_23     B_TEMP_33      K_WILSON      B_WILSON      FGCYCLES
   Iters          BOND         ANGLE      IMPROPER       TORSION    SINTORSION      TRIGONAL        PLANAR  BCORRELATION       CONTACT     IDEALDIST        CHIRAL    SIMILARITY       OCCSSUM  UTILDISTANCE     UTILANGLE   UTILTORSION        RHOFIT        ADP-01        ADP-02        ADP-03        ADP-04        ADP-05        ADP-06        ADP-07        ADP-08        ADP-09        ADP-10      RHOFITPC            QM
     100  4.433479E+05  4.019557E+05       41392.2       6.87620      0.107006       1.63559      0.200742      0.226420    0.00765591    0.00255525    0.00776070      0.994722       Opttag_D
     100       74.4280       1.76168       1.00000  0.000000E+00       1.00000  0.000000E+00      0.835557       41.5777       1.00000       43.8260  0.000000E+00       3.83930  0.000000E+00       3.83930  0.000000E+00  0.000000E+00      -7.67861       74.5490       2.25418        1       Scatag_D
     100       1691.14       5392.18  0.000000E+00  0.000000E+00       2209.63  0.000000E+00       3092.50       7862.48  0.000000E+00       10880.9       50.6556       10212.7 -1.000000E+10 -1.000000E+10 -1.000000E+10 -1.000000E+10 -1.000000E+10  0.000000E+00  0.000000E+00  0.000000E+00  0.000000E+00  0.000000E+00  0.000000E+00  0.000000E+00  0.000000E+00  0.000000E+00  0.000000E+00  0.000000E+00  0.000000E+00       Funtag_D
   Geometry Restraint Deviations SCREEN Information

 
 No -bcorrel_zeroocchydrogens_factor flag found - keeping default: will set B for OCC=0 hydrogens to    1.200 times heavy-atom B


 TLS information. Total number of groups:     2


 Group   Natoms    FixRB   FixTLS   Group Tag    Group Spec
     1     2768      Y        Y     tls1    { A|* }
     2     2770      Y        Y     tls2    { B|* }
 
NOTE BUSTER_TLS_SET tls1 { A|* }
NOTE BUSTER_TLS_T   tls1     -6.89402E-02  9.73396E-02  1.12143E-02 -5.14314E-02  3.70007E-02 -6.09601E-02
NOTE BUSTER_TLS_L   tls1      2.34061E+00  1.26720E+00  1.13030E+00 -7.76197E-01 -1.07598E+00  5.55382E-01
NOTE BUSTER_TLS_S   tls1      2.71047E-02 -3.50758E-01  5.52083E-01 -3.65020E-01  2.02419E-01 -2.02943E-01  6.79091E-03 -2.19839E-01
NOTE BUSTER_TLS_O   tls1     -2.32263E+01 -8.41675E+01  1.09752E+01
NOTE BUSTER_TLS_FIX tls1 ALL
 
NOTE BUSTER_TLS_SET tls2 { B|* }
NOTE BUSTER_TLS_T   tls2     -1.17934E-01 -4.34381E-02  3.08544E-02  1.57672E-02  3.71279E-02 -2.24165E-02
NOTE BUSTER_TLS_L   tls2      1.51304E+00  9.47989E-01  1.07060E+00  6.77124E-01 -9.34394E-01 -7.04506E-01
NOTE BUSTER_TLS_S   tls2     -4.36251E-03  8.11357E-02 -1.53271E-01  1.51330E-01  1.63309E-01  5.90040E-02 -7.47994E-02  3.57312E-02
NOTE BUSTER_TLS_O   tls2      2.22079E+01 -7.23190E+01  3.56420E+00
NOTE BUSTER_TLS_FIX tls2 ALL

 For final position, Geometry function summary
     term                           number     weight       rms        GooF      function     fn/numb
     ================================================================================================
     BOND bond lengths (angs)       5653       2.000      0.0078       0.387      1691.14       0.299
     ANGL bond angles (degs)        7670       2.000        0.99       0.593      5392.18       0.703
     TORS split-harmonic tors       1270       0.000       17.11       1.076         0.00       0.000
     SINTOR sinusoidal tors         1270       2.000       17.11                  2209.63       1.740
     PLAN general plane (angs)       963       5.000      0.0160       0.801      3092.50       3.211
     BCORrelations (angs^2)         5653      10.000       1.045       0.373      7862.48       1.391
     CONT (bad contacts)               0       5.000                                 0.00
     IDEAL(ideal-dist contact)      5159       4.000                             10880.89
     CHIRAL (gelly semiharmon)       740       5.000                                50.66
     SIM similarity restraint for NCS and/or TARGET                              10212.69
     OCCSUM restraint on sum of occupanies                                           0.00       as none defined
     DISTAN utility restraints interatomic distance                                  0.00       as none defined
     UTILANGLE utility angle restraints                                              0.00       as none defined
     UTILTOR utility restraints on torsion angles                                    0.00       as none defined
     Peptide omega torsion angles                           3.38 degs - monitor only
 
 WARNING: Have |delta/sigma| deviations > 5.0 sigma. Number of outliers for each term: 
 WARNING:         1 bond lengths. Worst is     5.7 sigs     1.46 Angs A|104:SD=CE (MET)
 WARNING:         2 bond angles.  Worst is     5.1 sigs   134.33 degs A|28:C.A(ASN)=A|29:N(ASN)=A|29:CA(ASN)

 Weighted rms      0.0078         0.995  (temporary rms bond and angle for BUSTER to pick up!)
 Weighted intra-residual rms(bond) for StdProtein (excluding H)      0.0076
 Weighted intra-residual rms(bond) for Buffer     (excluding H)      0.0031
 Outputing geometry progress graph in plotmtv format to file:Geom_Res.mtv

 ############### Running final geometry/screen analysis (screen_final.txt)

 s/r gelly normal termination
```

Statistics and scale factors vs. cycle number
```

|  |  |  |
| --- | --- | --- |
|  | - Rxpct factors- <Log-Lik Gain>- GEOM Residuals- Ln Sigma_A_ | - Scale Factors- B Factors- Imperfection Factors |

```
 Writing F_calc(model+solvent) to complete.mtz

   Final Geometry Information


---


### 2. Final results

Statistics and scale factors vs. cycle number
```

|  |  |  |
| --- | --- | --- |
|  | - Rxpct factors- <Log-Lik Gain>- GEOM Residuals- Ln Sigma_A_ | - Scale Factors- B Factors- Imperfection Factors |

```
   Average Figure of Merit
   Structure Factor Amplitudes


 # normal termination

  Program stopped.
```

closesok message: time spent in tree = 1744.99 (user) 41.00 (sys)

---

# Normal termination

```
 Resources usage summary:
     Time spent in client ...................  1744.99 (user)       41.02 (system)
     Time spent in scripts (    27 calls) ...     3.70 (user)        0.21 (system)
     Time spent in server ...................     0.00 (user)        0.01 (system)

     Max resident memory used (kb) .......... 475332
     Number of page faults .................. 0 (major)    975741 (minor)
     Number of context switches ............. 7576 (niced)    7182 (other)
```
